# Supplementary material for: Prediction of graft loss in living donor liver transplantation during the early postoperative period
Source: PLoS Comput Biol. 2025 Nov 24;21(11):e1013734. doi: 10.1371/journal.pcbi.1013734 (PMC12671733; doi:10.1371/journal.pcbi.1013734)
Supplement: S1 Text — Fig A. Sensitivity analysis of Random Forest. Fig B. Calibration plot and decision curve analysis for early graft loss prediction. Fig C. Early graft loss prediction without donor information. Fig D. Comparison of the important features between groups with early, intermediate, and late or no graft loss. Fig E. Comparison of important features between all groups. Table A. Comparison of clinical data between the derivation and validation datasets. Table B. Comparison of derivation data in each group. Table C. Comparison of clinical data in each group. Table D. Comparison of Generalized Wilcoxon test results with Benjamini-Hochberg correction in derivation cohort data. Table E. Comparison of Bonferroni-corrected Wilcoxon rank-sum test results. Table F. Comparison of Bonferroni-corrected Fisher’s exact test results. Table G. Comparison of Generalized Wilcoxon test results with Benjamini-Hochberg correction in group-annotated validation cohort data. (DOCX) [file pcbi.1013734.s001.docx]

**Supplementary Information**

Prediction of graft loss in living donor liver transplantation in early postoperative period

Raiki Yoshimura^1,$^, Naotoshi Nakamura^1,2,$^, Takeru Matsuura^1,$^, Takeo Toshima^3^, Takasuke Fukuhara^4^, Kazuyuki Aihara^5^, Katsuhito Fujiu^6,7^, Shingo Iwami^1,5,8,9,10,11,12,&,*^ and Tomoharu Yoshizumi^3,&,*^

^1^interdisciplinary Biology Laboratory (iBLab), Division of Natural Science, Graduate School of Science, Nagoya University, Nagoya, Japan. ^2^Department of Data Science, Yokohama City University, Yokohama, Japan ^3^Department of Surgery and Science, Graduate School of Medical Sciences, Kyushu University, Fukuoka, Japan. ^4^Department of Virology, Faculty of Medical Sciences, Kyushu University, Fukuoka, Japan. ^5^International Research Center for Neurointelligence, The University of Tokyo Institutes for Advanced Study, The University of Tokyo, Tokyo, Japan. ^6^Department of Cardiovascular Medicine, Graduate School of Medicine, The University of Tokyo, Tokyo, Japan. ^7^Department of Integrative Physiology, Institute of Science Tokyo, Tokyo, Japan. ^8^Institute of Mathematics for Industry, Kyushu University, Fukuoka, Japan. ^9^Institute for the Advanced Study of Human Biology (ASHBi), Kyoto University, Kyoto, Japan. ^10^Interdisciplinary Theoretical and Mathematical Sciences Program (iTHEMS), RIKEN, Saitama, Japan. ^11^NEXT-Ganken Program, Japanese Foundation for Cancer Research (JFCR), Tokyo, Japan. ^12^Science Groove Inc., Fukuoka, Japan.


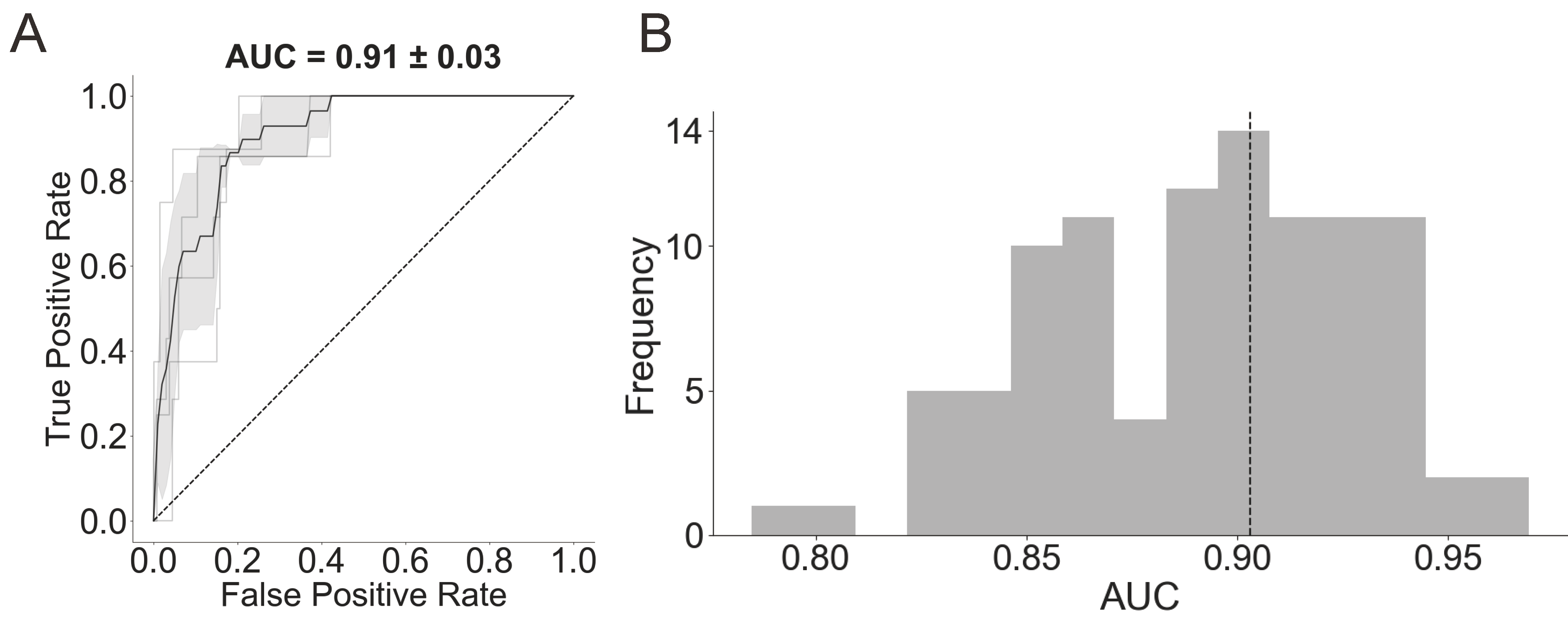


**Fig A |** **Sensitivity analysis of Random Forest:** (A) The AUCs calculated by ROCs of the RF classifiers trained to predict early graft loss using 4-fold cross-validation in derivation cohort. The corresponding ROC-AUC is calculated and displayed at the top of the panel. (B) The distribution of ROC-AUCs of the RF classifiers, based on 100 different datasets with different seeds for splitting derivation and validation data, is shown. The average and standard deviation of the ROC-AUCs is $0.90\pm004$ and the vertical dashed line is the corresponding ROC-AUC in **Fig 1C.**


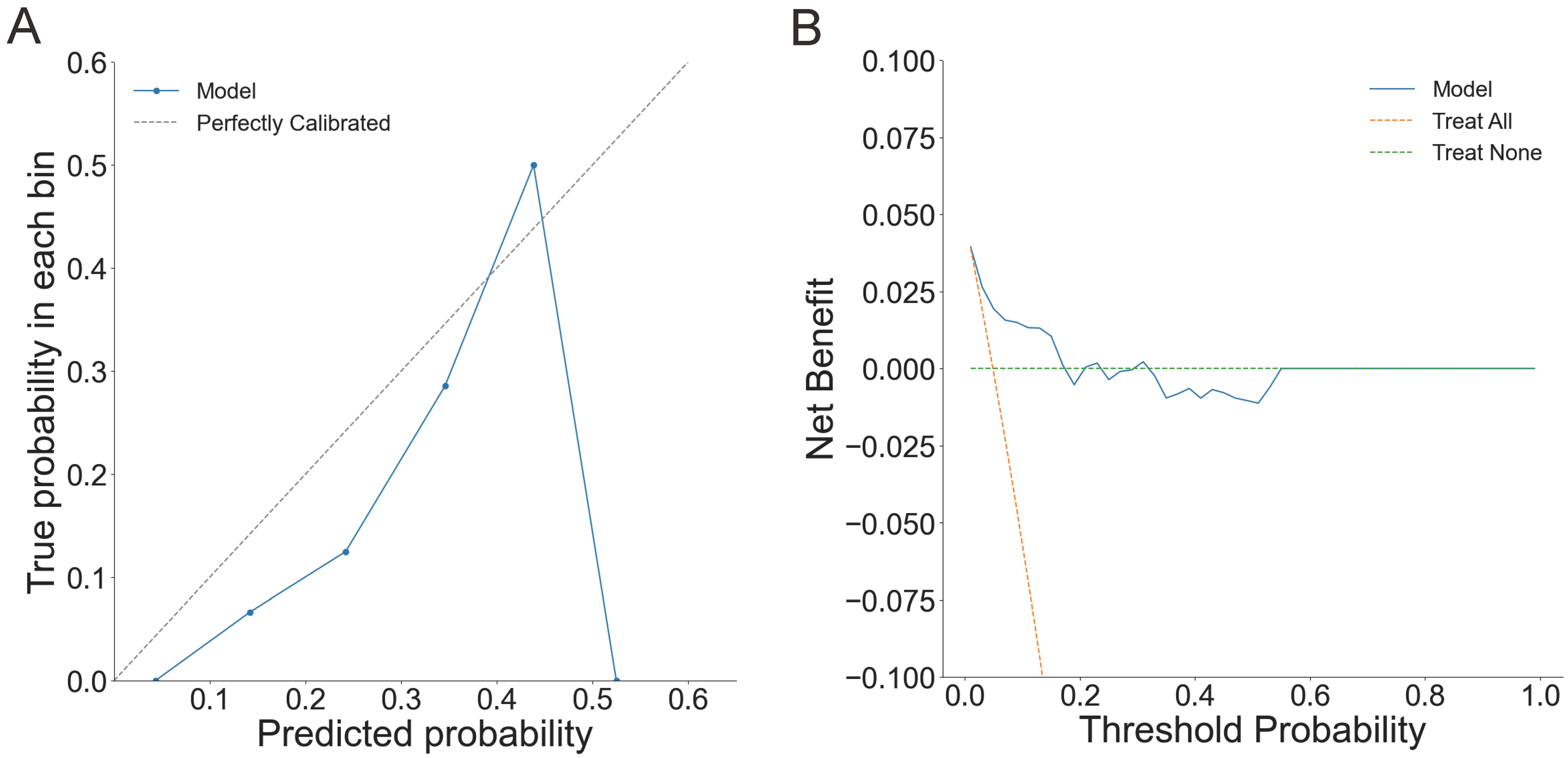


**Fig B | Calibration plot and decision curve analysis for early graft loss prediction: (A)**The blue line indicates the observed event frequency, and the gray line indicates perfect calibration. The model slightly underestimates risk overall, with a sharper decline at the upper end due to the small number of cases with predicted probabilities >0.5. **(B)**The orange dotted line represents the strategy of treating all patients; the blue dotted line corresponds to treatment based on the model's predicted probabilities; and the green dotted line indicates the strategy of treating no patients. The net benefit of treating all patients crosses zero, while the model maintains a positive net benefit of over 0.01 within the threshold probability range of 0.05 to 0.15.

**
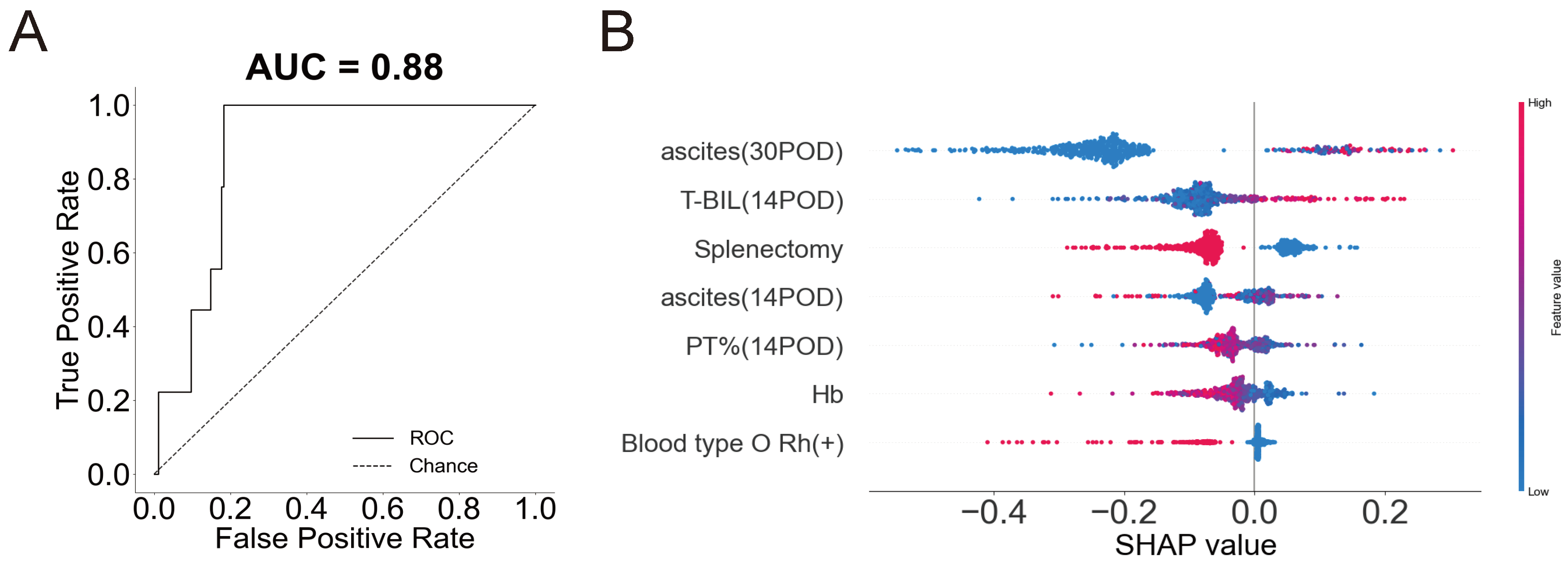
**

**Fig C | Early graft loss prediction without donor information: (A)** The ROC curve of RF classifiers trained to predict early graft loss without donor information. The corresponding ROC-AUC is calculated and displayed at the top of the panel. **(B)** Feature importance of the predictive model with the highest ROC-AUC value is illustrated as a SHAP summary plot.

**
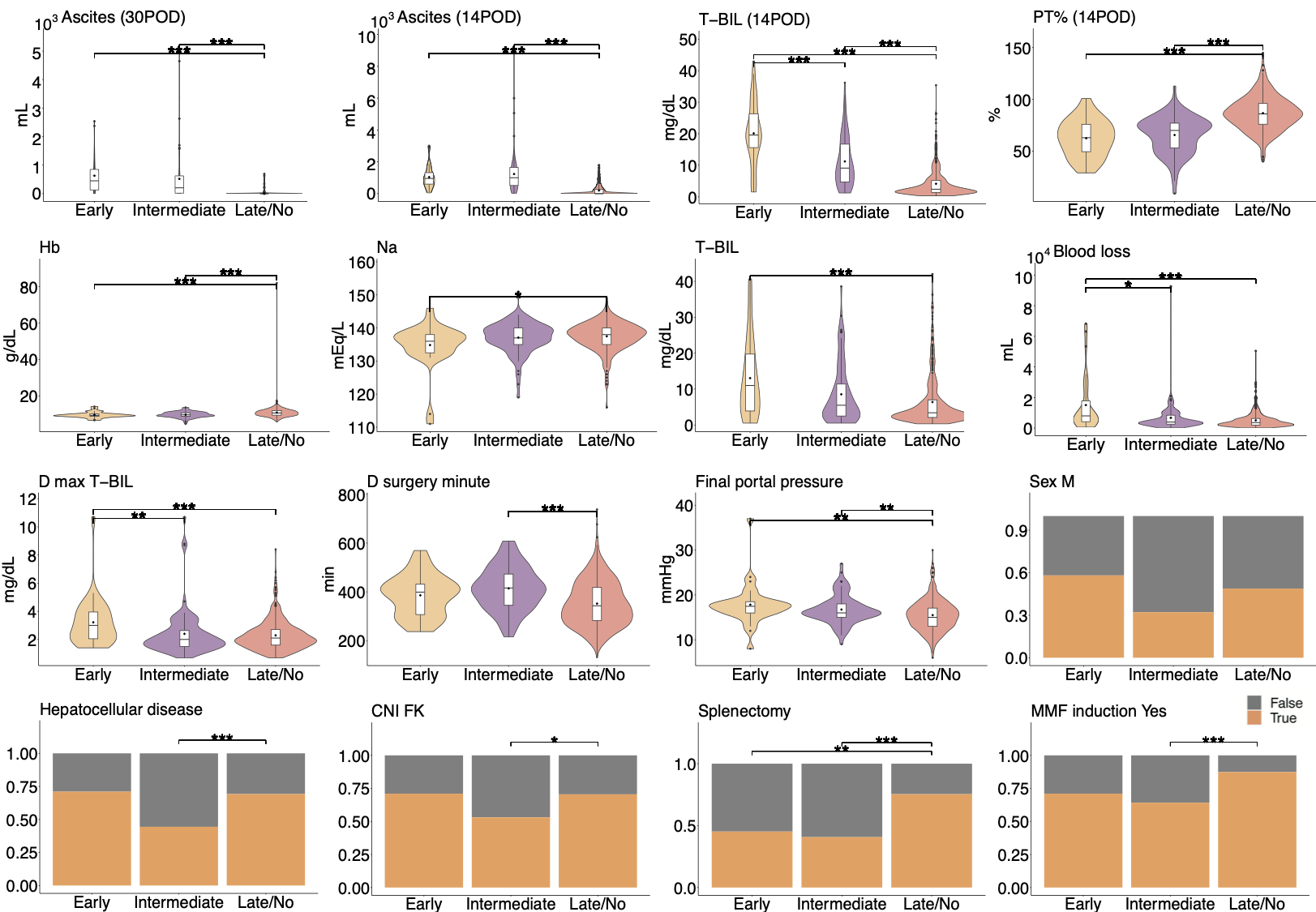
 Fig D | Comparison of the important features between groups with early, intermediate, and late or no graft loss:** The preoperative, intraoperative, and postoperative data selected as the important factors by SHAP for non-early graft loss prediction shown in **Fig 2E** are plotted and colored accordingly. Statistical significance is calculated using the Wilcoxon rank sum test for continuous values and p-values are expressed as follows (N.S.: p-value > 0.05, *: p-value ≤ 0.05, **: p-value ≤ 0.01, and ***: p-value ≤ 0.001, respectively). Also, Fisher’s exact test for categorical values is used and whether there were significant differences in the proportions of each group relative to the total was examined (N.S.: p-value > 0.05, *: p-value ≤ 0.05, **: p-value ≤ 0.01, and ***: p-value ≤ 0.001, respectively). "TRUE" indicates that the item is applicable. For example, in the context of Sex M, "TRUE" means cases who are men, and "FALSE" means cases who are women. Orange represents the percentage of “TRUE” and gray represents the percentage of “FALSE”.

**
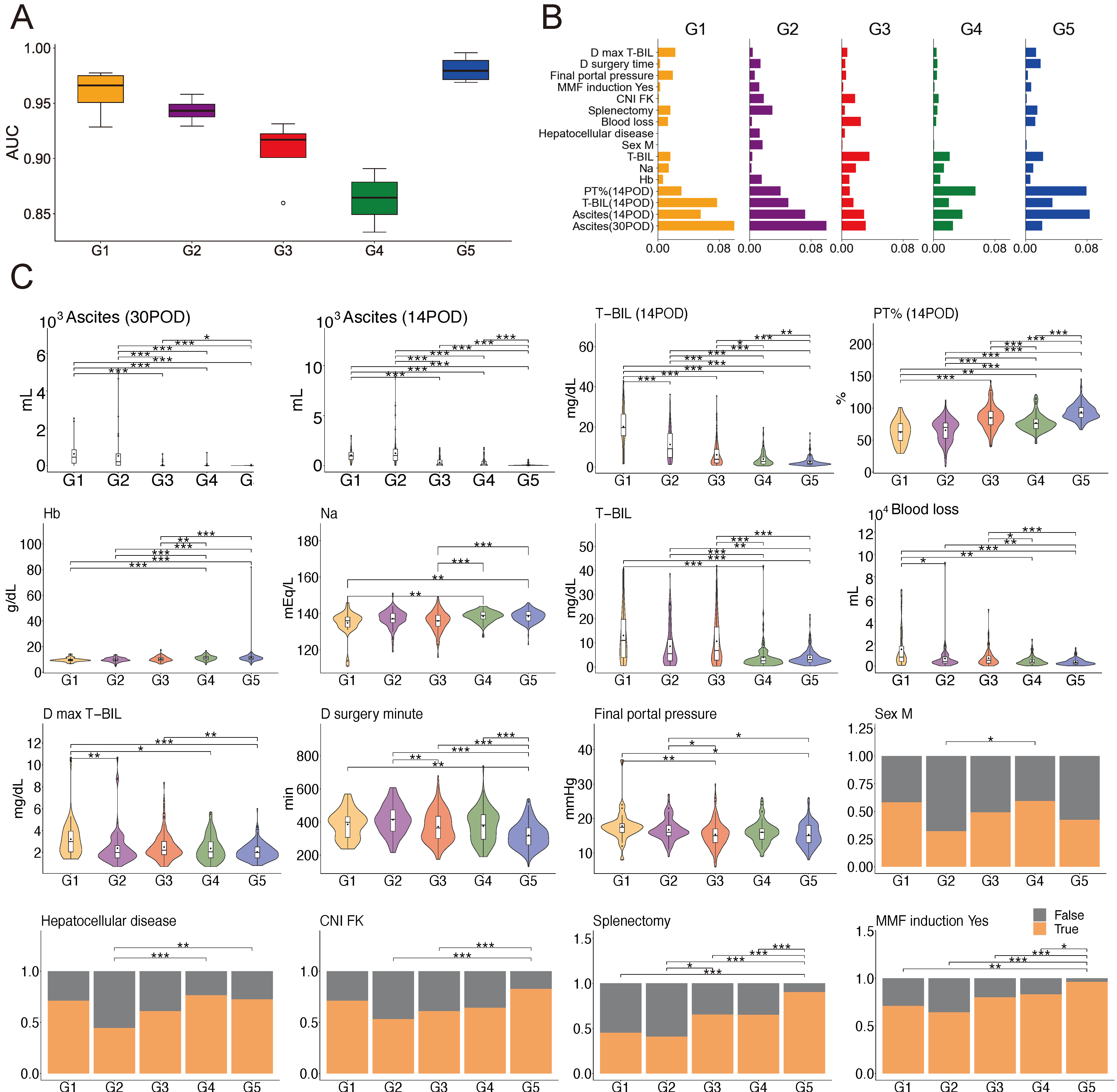
**

**Fig E | Comparison of important features between all groups: (A)** The AUCs calculated by ROCs of the RF classifiers trained to predict whether each patient belongs to each group as a binary classification (e.g., either G1 or not) are plotted and colored accordingly. In each binary prediction, five AUC values were obtained, and quartiles were calculated by using 5-fold cross-validation. **(B)** Feature importance of the predictive model in **A** is illustrated as a bar plot (note that the panels for the feature importance of G1 and G2 are identical to those in **Fig 2E**). The *x*-axis in each variable represents mean SHAP value. Abbreviations and definitions: 30POD, 30 postoperative days; 14POD, 14 postoperative days; ascites, ascites volume; T-BIL, serum total bilirubin; PT%, prothrombin time activity percentage; Na, sodium; Hb, hemoglobin; blood loss, blood loss during surgery; splenectomy, the spleen was removed during surgery; hepatocellular disease, diagnosis on admission to hospital; CNI FK, calcineurin inhibitor was FK506 (tacrolimus); MMF induction yes, mycophenolate mofetil at the time of introduction; final portal pressure, portal pressure measured at the end of surgery; D surgery time, the time of donor surgery; D max T-BIL, donor's serum total bilirubin. **(C)** The preoperative, intraoperative, and postoperative data selected as the important factors by SHAP for non-early graft loss prediction shown in **B** are plotted and colored accordingly. Statistical significance is calculated using the Wilcoxon rank sum test for continuous values and p-values are expressed as follows (N.S.: p-value > 0.05, *: p-value ≤ 0.05, **: p-value ≤ 0.01, and ***: p-value ≤ 0.001, respectively). Also, Fisher’s exact test for categorical values is used and whether there were significant differences in the proportions of each group relative to the total was examined (N.S.: p-value > 0.05, *: p-value ≤ 0.05, **: p-value ≤ 0.01, and ***: p-value ≤ 0.001, respectively). "TRUE" indicates that the item is applicable. For example, in the context of precondition home, "TRUE" means cases who were homebound before LDLT, and "FALSE" means cases who were hospitalized before LDLT. Orange represents the percentage of “TRUE” and gray represents the percentage of “FALSE”.

**Table A | Comparison of clinical data between the derivation and validation datasets**

| **Numeric data** |  | **Derivation (N = 563)** | **Validation (N = 185)** | **All (N = 748)** |  |
| --- | --- | --- | --- | --- | --- |
| **Recipients** |  |  |  |  |  |
| **Preoperative** |  |  |  |  | ***p*-value**^†^ |
| Mono | % | 8.2 (3.1) | 8.2 (3.3) | 8.2 (3.2) | 𝑁.𝑆. |
| NLR | − | 4.4 (5.8) | 4.4 (6.3) | 4.4 (5.9) | ∗∗ |
| LMR | − | 3.1 (1.8) | 3.2 (2.1) | 3.2 (1.9) | 𝑁.𝑆. |
| Age | years | 53.8 (11.6) | 53.6 (12.4) | 53.7 (11.8) | 𝑁.𝑆. |
| Height | cm | 160.7 (8.7) | 159.8 (8.2) | 160.5 (8.6) | 𝑁.𝑆. |
| Weight | kg | 61.8 (11.5) | 59.3 (10.1) | 61.2 (11.2) | ∗ |
| BMI | kg/m^2^ | 23.9 (3.6) | 23.2 (3.2) | 23.7 (3.5) | 𝑁.𝑆. |
| BSA | m^2^ | 1.7 (0.2) | 1.6 (0.2) | 1.6 (0.2) | ∗ |
| T-BIL | mg/dL | 7.1 (7.9) | 7.7 (9.0) | 7.2 (8.2) | 𝑁.𝑆. |
| Albumin | g/dL | 2.9 (0.5) | 3.0 (1.7) | 2.9 (0.9) | 𝑁.𝑆. |
| PT | % | 52.0 (16.7) | 53.3 (17.7) | 52.3 (16.9) | 𝑁.𝑆. |
| APTT | sec | 45.7 (14.2) | 44.8 (14.3) | 45.5 (14.2) | 𝑁.𝑆. |
| BUN | mg/dL | 17.7 (13.3) | 16.3 (12.4) | 17.4 (13.1) | ∗∗ |
| Cr | mg/dL | 0.8 (0.6) | 0.8 (0.4) | 0.8 (0.6) | 𝑁.𝑆. |
| GFR | mL/min | 82.1 (35.4) | 84.8 (36.8) | 82.8 (35.7) | 𝑁.𝑆. |
| Na | mEq/L | 137.3 (4.6) | 137.5 (4.1) | 137.3 (4.5) | 𝑁.𝑆. |
| WBC | 10^3^/μL | 4.7 (3.0) | 4.9 (3.1) | 4.8 (3.0) | 𝑁.𝑆. |
| PLT | 10^4^/μL | 8.7 (6.4) | 8.7 (6.5) | 8.7 (6.5) | 𝑁.𝑆. |
| Hb | g/dL | 10.7 (3.6) | 11.5 (8.2) | 10.9 (5.1) | 𝑁.𝑆. |
| **During surgery** |  |  |  |  |  |
| Surgery time | min | 753.0 (155.1) | 737.3 (157.1) | 749.1 (155.7) | 𝑁.𝑆. |
| Blood loss | 10^3^ mL | 5.9 (7..7) | 7.1 (14.8) | 6.2 (9.9) | 𝑁.𝑆. |
| Warm ischemic time | min | 42.3 (12.9) | 41.1 (12.2) | 42.0 (12.7) | 𝑁.𝑆. |
| Cold ischemic time | min | 102.9 (58.4) | 104.5 (55.5) | 103.3 (57.6) | 𝑁.𝑆. |
| Portal blood flow/GW | − | 3.3 (1.3) | 3.3 (1.3) | 3.3 (1.3) | 𝑁.𝑆. |
| Post-reperfusion portal pressure | mmHg | 19.0 (4.5) | 18.9 (5.4) | 19.0 (4.8) | 𝑁.𝑆. |
| Final portal pressure | mmHg | 15.8 (3.6) | 15.4 (3.7) | 15.7 (3.6) | 𝑁.𝑆. |
| **After surgery** |  |  |  |  |  |
| T-BIL (14POD) | mg/dL | 6.0 (7.0) | 5.5 (6.2) | 5.9 (6.8) | 𝑁.𝑆. |
| Ascites (14POD) | mL | 391.4 (726.1) | 464.8 763.0) | 409.6 (735.5) | 𝑁.𝑆. |
| Ascites (30POD) | mL | 113.5 (420.7) | 107.6 (366.4) | 112.0 (407.7) | 𝑁.𝑆. |
| PT (14POD) | % | 82.3 (18.8) | 83.7 (17.8) | 82.7 (18.6) | 𝑁.𝑆. |
| **Donors/grafts** |  |  |  |  |  |
| **Preoperative** |  |  |  |  |  |
| Age | years | 38.1 (11.0) | 37.8 (11.0) | 38.0 (11.0) | 𝑁.𝑆. |
| Height | cm | 166.1 (8.3) | 166.1 (8.3) | 166.1 (8.3) | 𝑁.𝑆. |
| Weight | kg | 61.8 (10.2) | 60.6 (9.5) | 61.5 (10.1) | 𝑁.𝑆. |
| BSA | m^2^ | 1.7 (0.2) | 1.7 (0.2) | 1.7 (0.2) | 𝑁.𝑆. |
| BMI | kg/m^2^ | 22.3 (2.6) | 21.9 (2.4) | 22.2 (2.6) | 𝑁.𝑆. |
| Total liver volume | 10^2^ mL | 11.3 (2.0) | 11.2 (1.8) | 11.2 (1.9) | 𝑁.𝑆. |
| Donor/recipient weight ratio | − | 1.0 (0.2) | 1.1 (0.3) | 1.0 (0.2) | 𝑁.𝑆. |
| Predict GV/SLV | % | 46.9 (10.2) | 46.2 (11.3) | 46.7 (10.4) | 𝑁.𝑆. |
| **During surgery** |  |  |  |  |  |
| Blood loss | mL | 401.7 (366.5) | 412.7 (393.5) | 404.4 (373.1) | 𝑁.𝑆. |
| Surgery time | min | 362.7 (93.9) | 361.3 (100.9) | 362.3 (95.6) | 𝑁.𝑆. |
| Expected residual liver | % | 55.1 (12.5) | 56.0 (12.4) | 55.3 (12.5) | 𝑁.𝑆. |
| Graft weight | g | 489..9 (120.3) | 474.4 (109.8) | 486.1 (117.9) | 𝑁.𝑆. |
| GV/SLV | % | 42.1 (9.2) | 41.7 (9.2) | 42.0 (9.2) | 𝑁.𝑆. |
| GRWR | − | 0.8 (0.2) | 0.8 (0.2) | 0.8 (0.2) | 𝑁.𝑆. |
| **After surgery** |  |  |  |  |  |
| Max T-BIL | mg/dL | 2.3 (1.1) | 2.3 (1.1) | 2.3 (1.2) | 𝑁.𝑆. |
| Max ALT | U/L | 499.1 (249.8) | 499.8 (275.1) | 499.7 (254.8) | 𝑁.𝑆. |

†: $N.S. :p>0.05$, $* :p\leq0.05$, $** :p\leq0.01$, $*** :p\leq0.001$

| **Categorical data** | **Derivation (N = 563)** | **Validation (N = 185)** | **All (N = 748)** |  |
| --- | --- | --- | --- | --- |
| **Recipients** |  |  |  |  |
| **Preoperative** |  |  |  | ***p*-value**^†^ |
| Diagnosis |  |  |  | 𝑁.𝑆. |
| Hepatocellular disease | 65.7% | 62.2% | 64.8% |  |
| Cholestasis | 21.3% | 23.2% | 21.8% |  |
| Acute liver failure | 9.1% | 11.4% | 9.6% |  |
| Hepatocellular disease + cholestasis | 0.2% | 0.0% | 0.1% |  |
| Metabolic disease | 0.2% | 0.0% | 0.1% |  |
| Vascular disease | 0.4% | 0.5% | 0.4% |  |
| Other | 3.2% | 2.7% | 3.1% |  |
| HCC |  |  |  | 𝑁.𝑆. |
| Yes | 35.3% | 36.8% | 35.7% |  |
| Sex |  |  |  | ∗ |
| M | 46.9% | 38.4% | 44.8% |  |
| F | 53.1% | 61.6% | 55.2% |  |
| Sex combination |  |  |  | ∗ |
| Man to man | 27.9% | 18.9% | 25.7% |  |
| Man to woman | 34.8% | 45.9% | 37.6% |  |
| Woman to woman | 18.3% | 15.7% | 17.6% |  |
| Woman to man | 19.0% | 19.5% | 19.1% |  |
| HBsAb |  |  |  | ∗ |
| Yes | 22.2% | 15.1% | ﻿20.5% |  |
| (+) | 0.0% | 0.5% | 0.1% |  |
| ND | 0.4% | 1.1% | 0.5% |  |
| HTLV1 |  |  |  | 𝑁.𝑆. |
| Yes | 5.3% | 5.4% | ﻿5.3% |  |
| HCVAB |  |  |  |  |
| Yes | 31.1% | 27.6% | ﻿30.2% |  |
| Yes(RNA-) | 2.3% | 2.2% | ﻿2.3% |  |
| Precondition |  |  |  | 𝑁.𝑆. |
| Home | 62.7% | 56.2% | 61.1% |  |
| Hospitalized | 26.5% | 29.7% | 27.3% |  |
| ICU | 10.5% | 13.5% | 11.2% |  |
| Relationship |  |  |  | 𝑁.𝑆. |
| Son | 37.5% | 37.8% | 37.6% |  |
| Daughter | 19.2% | 19.5% | 19.3% |  |
| Wife | 8.5% | 8.1% | 8.4% |  |
| Sister | 6.2% | 3.2% | 5.5% |  |
| Brother | 9.6% | 8.1% | 9.2% |  |
| Nephew | 0.9% | 3.8% | 1.6% |  |
| Husband | 9.4% | 9.7% | 9.5% |  |
| Parents | 4.6% | 7.6% | 5.3% |  |
| Cousin | 0.4% | 0.5% | 0.4% |  |
| Other | 3.4% | 1.1% | 2.8% |  |
| Aunt | 0.2% | 0.0% | 0.1% |  |
| Niece | 0.0% | 0.5% | 0.1% |  |
| Blood relative |  |  |  | 𝑁.𝑆. |
| Yes | 78.7% | 80.5% | 79.1% |  |
| Blood type |  |  |  | 𝑁.𝑆. |
| AB Rh(+) | 12.8% | 10.3% | 12.2% |  |
| O Rh(+) | 26.8% | 27.0% | 26.9% |  |
| B Rh(+) | 21.3% | 20.5% | 21.1% |  |
| A Rh(+) | 38.7% | 40.5% | 39.2% |  |
| A Rh(-) | 0.4% | 1.6% | 0.7% |  |
| ABO |  |  |  | 𝑁.𝑆. |
| Compatible | 23.1% | 21.6% | 22.7% |  |
| Identical | 61.1% | 64.3% | 61.9% |  |
| Incompatible | 15.8% | 14.1% | 15.4% |  |
| Portal vein thrombosis |  |  |  | 𝑁.𝑆. |
| Yes | 16.9% | 15.1% | 16.4% |  |
| DM |  |  |  | 𝑁.𝑆. |
| Yes | 18.5% | 14.1% | 17.4% |  |
| Refractory ascites |  |  |  | 𝑁.𝑆. |
| Yes | 67.7% | 65.4% | 67.1% |  |
| None | 27.5% | 31.4% | 28.5% |  |
| CKD |  |  |  | 𝑁.𝑆. |
| Yes | 19.4% | 18.9% | 19.3% |  |
| Child class |  |  |  | 𝑁.𝑆. |
| Child A | 4.3% | 4.3% | 4.3% |  |
| Child B | 17.9% | 22.7% | 19.1% |  |
| Child C | 68.4% | 60.5% | 66.4% |  |
| FHF | 9.1% | 11.4% | 9.6% |  |
| **During surgery** |  |  |  |  |
| Portal modulation |  |  |  | 𝑁.𝑆. |
| Splenic artery ligation | 7.3% | 6.5% | 7.1% |  |
| Splenectomy | 68.7% | 67.0% | 68.3% |  |
| None | 23.6% | 25.4% | 24.1% |  |
| History of upper abdominal laparotomy |  |  |  | 𝑁.𝑆. |
| Yes | 18.8% | 22.2% | 19.7% |  |
| Liver resection or liver transplant history |  |  |  | 𝑁.𝑆. |
| Yes | 3.9% | 4.3% | 4.0% |  |
| Esophageal varices |  |  |  | 𝑁.𝑆. |
| Yes(treated) | 30.4% | 25.4% | 29.1% |  |
| Yes | 19.5% | 23.2% | 20.5% |  |
| Portocaval shunt (>10mm) |  |  |  | 𝑁.𝑆. |
| None | 61.3% | 57.3% | 60.3% |  |
| Yes and ligated | 38.2% | 41.6% | 39.0% |  |
| No ligation | 0.2% | 0.0% | 0.1% |  |
| Veno-venous bypass |  |  |  | 𝑁.𝑆. |
| Yes | 9.1% | 5.4% | ﻿8.2% |  |
| Biliary reconstruction |  |  |  | 𝑁.𝑆. |
| Roux-en Y | 13.0% | 15.1% | 13.5% |  |
| Duct-to-duct | 86.5% | 83.8% | 85.8% |  |
| Bile duct suture method |  |  |  | 𝑁.𝑆. |
| Interrupted + continuous | 19.5% | 17.8% | 19.1% |  |
| Interrupted | 76.2% | 74.6% | 75.8% |  |
| Continuous | 3.7% | 5.9% | 4.3% |  |
| Biliary stent |  |  |  | 𝑁.𝑆. |
| External fistula | 94.8% | 91.9% | 94.1% |  |
| Internal | 1.4% | 3.2% | 1.9% |  |
| **After surgery** |  |  |  |  |
| CNI |  |  |  | 𝑁.𝑆. |
| CYA | 20.6% | 19.5% | 20.3% |  |
| FK | 68.0% | 66.5% | 67.6% |  |
| FK→CYA | 6.2% | 10.3% | 7.2% |  |
| CYA→FK | 2.7% | 0.5% | 2.1% |  |
| MMF induction |  |  |  | 𝑁.𝑆. |
| Yes | 83.1% | 81.6% | 82.8% |  |
| Simulect |  |  |  | 𝑁.𝑆. |
| Yes | 2.3% | 2.2% | 2.3% |  |
| Bile leak |  |  |  | 𝑁.𝑆. |
| Yes | 8.5% | 7.6% | 8.3% |  |
| **Donors / grafts** |  |  |  |  |
| **Preoperative** |  |  |  |  |
| HBcAb |  |  |  | 𝑁.𝑆. |
| Yes | 12.8% | 9.2% | 11.9% |  |
| HTLV1 |  |  |  | 𝑁.𝑆. |
| Yes | 1.4% | 0.5% | 1.2% |  |
| Sex |  |  |  | 𝑁.𝑆. |
| M | 62.7% | 64.9% | 63.2% |  |
| F | 37.3% | 35.1% | 36.8% |  |
| Technique |  |  |  | 𝑁.𝑆. |
| Right lobectomy | 48.1% | 45.4% | 47.5% |  |
| Extended left lobectomy + caudate lobectomy | 42.5% | 44.9% | 43.0% |  |
| Extended left lobectomy | 3.2% | 3.8% | 3.3% |  |
| Left lobectomy + caudate lobectomy | 3.7% | 3.8% | 3.7% |  |
| Extended posterior segmentectomy | 2.0% | 1.1% | 1.7% |  |
| Posterior segmentectomy | 0.2% | 0.5% | 0.3% |  |
| Extended right lobectomy | 0.4% | 0.5% | 0.4% |  |
| Blood type |  |  |  | 𝑁.𝑆. |
| A Rh(+) | 36.6% | 44.9% | 38.6% |  |
| A Rh(-) | 0.2% | 0.5% | 0.3% |  |
| O Rh(+) | 35.9% | 35.7% | 35.8% |  |
| O Rh(-) | 0.4% | 0.0% | 0.3% |  |
| B Rh(+) | 17.8% | 11.9% | 16.3% |  |
| AB Rh(+) | 9.2% | 7.0% | 8.7% |  |
| **During surgery** |  |  |  |  |
| Skin incision |  |  |  | 𝑁.𝑆. |
| Benz | 35.7% | 36.8% | 36.0% |  |
| Midline | 49.4% | 53.5% | 50.4% |  |
| Reverse L | 14.4% | 9.2% | 13.1% |  |
| Midline lap auxiliary | 0.2% | 0.0% | ﻿0.1% |  |
| **After surgery** |  |  |  |  |
| Complication CD class |  |  |  | 𝑁.𝑆. |
| I | 13.1% | 10.8% | 12.6% |  |
| II | 8.3% | 9.2% | 8.6% |  |
| IIIa | 4.6% | 3.8% | 4.4% |  |
| IIIb | 1.6% | 2.7% | 1.9% |  |
| No | 72.1% | 73.5% | 72.5% |  |
| Graft loss | 22.9% | 25.9% | 23.7% |  |

†: $N.S. :p>0.05$, $* :p\leq0.05$, $** :p\leq0.01$, $*** :p\leq0.001$

**Table B | Comparison of derivation data in each group**

| **Clinical data** | **Unit** | **G1 (N=31)** | **G2 (N=81)** | **G3 (N=161)** | **G4 (N=106)** | **G5 (N=184)** | **Derivation (N=563)** | **Kruskal-Wallis** |
| --- | --- | --- | --- | --- | --- | --- | --- | --- |
| **Numeric data** |  |  |  |  |  |  |  |  |
| **Recipients** |  |  |  |  |  |  |  |  |
| **Preoperative** |  |  |  |  |  |  |  | ***p*-value**^†^ |
| Mono | % | 8.8 (3.6) | 7.8 (3.2) | 8.5 (3.5) | 8.1 (3.3) | 8.1 (2.6) | 8.2 (3.1) | 𝑁.𝑆. |
| NLR | − | 6.4 (6.0) | 5.3 (6.6) | 7.0 (8.3) | 2.2 (1.5) | 2.8 (2.3) | 4.4 (5.8) | ∗∗∗ |
| LMR | − | 2.5 (2.3) | 2.9 (1.7) | 2.2 (1.5) | 4.1 (1.6) | 3.6 (1.7) | 3.1 (1.8) | ∗∗∗ |
| Age | years | 53.7 (10.4) | 49.7 (13.5) | 53.4 (12.3) | 54.0 (11.3) | 55.7 (10.0) | 53.8 (11.6) | ∗ |
| Height | cm | 161.6 (8.5) | 158.8 (8.0) | 160.8 (8.7) | 162.6 (9.3) | 160.1 (8.4) | 160.7 (8.7) | ∗ |
| Weight | kg | 63.8 (12.6) | 57.6 (10.4) | 61.9 (11.9) | 64.0 (11.3) | 61.9 (11.1) | 61.8 (11.5) | ∗∗ |
| BMI | kg/m^2^ | 24.5 (4.9) | 22.8 (3.7) | 23.9 (3.9) | 24.1 (3.1) | 24.1 (3.3) | 23.9 (3.6) | ∗ |
| BSA | m^2^ | 1.7 (0.2) | 1.6 (0.2) | 1.7 (0.2) | 1.7 (0.2) | 1.7 (0.2) | 1.7 (0.2) | ∗∗ |
| T-BIL | mg/dL | 13.0 (11.2) | 8.5 (8.4) | 10.7 (9.7) | 4.0 (5.1) | 4.0 (3.3) | 7.1 (7.9) | ∗∗∗ |
| Albumin | g/dL | 2.8 (0.6) | 2.9 (0.6) | 2.9 (0.5) | 2.9 (0.6) | 2.8 (0.5) | 2.9 (0.5) | 𝑁.𝑆. |
| PT | % | 46.2 (19.1) | 47.9 (17.0) | 45.6 (14.7) | 56.9 (17.1) | 57.5 (14.9) | 52.0 (16.7) | ∗∗∗ |
| APTT | sec | 50.8 (18.1) | 48.1 (19.7) | 49.6 (16.7) | 43.7 (9.6) | 41.5 (7.6) | 45.7 (14.2) | ∗∗∗ |
| BUN | mg/dL | 24.4 (19.7) | 19.7 (13.6) | 22.2 (18.1) | 14.5 (7.1) | 13.6 (5.9) | 17.7 (13.3) | ∗∗∗ |
| Cr | mg/dL | 1.0 (1.1) | 0.9 (0.9) | 1.0 (0.8) | 0.7 (0.3) | 0.7 (0.3) | 0.8 (0.6) | ∗∗∗ |
| GFR | mL/min | 82.6 (52.3) | 78.4 (37.9) | 75.2 (37.3) | 87.8 (33.9) | 86.3 (28.4) | 82.1 (35.4) | ∗∗∗ |
| Na | mEq/L | 134.8 (6.9) | 137.1 (4.8) | 135.9 (5.5) | 138.4 (3.1) | 138.4 (3.2) | 137.3 (4.6) | ∗∗∗ |
| WBC | 10^3^/μL | 4540.0 (2561.8) | 5669.1 (3653.2) | 5739.2 (3616.8) | 3645.1 (1815.5) | 4092.1 (2220.7) | 4730.5 (2995.3) | ∗∗∗ |
| PLT | 10^4^/μL | 8.0 (4.7) | 8.8 (6.9) | 8.3 (5.9) | 8.6 (7.1) | 9.3 (6.6) | 8.7 (6.4) | 𝑁.𝑆. |
| Hb | g/dL | 9.6 (1.6) | 9.8 (1.6) | 10.3 (1.9) | 11.2 (1.9) | 11.4 (5.5) | 10.7 (3.6) | ∗∗∗ |
| **During surgery** |  |  |  |  |  |  |  |  |
| Surgery time | min | 821.2 (189.7) | 790.8 (183.3) | 759.6 (145.8) | 771.9 (166.6) | 708.2 (123.0) | 753.0 (155.1) | ∗∗∗ |
| Blood loss | 10^3^ mL | 15034.1 (18087.0) | 6728.4 (10711.8) | 7045.5 (6866.0) | 4649.8 (3952.0) | 3636.8 (2810.5) | 5874.7 (7730.2) | ∗∗∗ |
| Warm ischemic time | min | 44.7 (12.1) | 39.0 (11.3) | 42.5 (12.3) | 41.8 (14.0) | 43.3 (13.3) | 42.3 (12.9) | ∗ |
| Cold ischemic time | min | 111.1 (56.7) | 86.2 (63.4) | 104.7 (63.8) | 102.5 (56.3) | 107.4 (51.3) | 102.9 (58.4) | ∗∗∗ |
| Portal blood flow/GW | − | 3.2 (1.5) | 3.2 (1.3) | 3.4 (1.4) | 3.2 (1.2) | 3.5 (1.3) | 3.3 (1.3) | 𝑁.𝑆. |
| Post-reperfusion portal pressure | mmHg | 18.8 (5.2) | 19.4 (3.4) | 18.3 (4.8) | 19.1 (4.3) | 19.4 (4.6) | 19.0 (4.5) | ∗ |
| Final portal pressure | mmHg | 17.8 (4.7) | 16.7 (3.2) | 15.4 (3.7) | 16.0 (3.3) | 15.3 (3.3) | 15.8 (3.6) | ∗∗∗ |
| **After surgery** |  |  |  |  |  |  |  |  |
| T-BIL (14POD) | mg/dL | 20.1 (10.4) | 11.2 (7.9) | 6.0 (5.9) | 3.9 (3.6) | 2.6 (2.4) | 6.0 (7.0) | ∗∗∗ |
| Ascites (14POD) | mL | 1036.9 (708.3) | 1234.0 (1321.9) | 318.0 (409.9) | 288.6 (402.5) | 35.3 (99.8) | 391.4 (726.1) | ∗∗∗ |
| Ascites (30POD) | mL | 628.4 (641.7) | 517.2 (861.5) | 10.1 (61.0) | 7.8 (67.7) | 0.4 (4.5) | 113.5 (420.7) | ∗∗∗ |
| PT (14POD) | % | 62.4 (17.9) | 65.6 (18.0) | 84.8 (18.1) | 77.0 (13.4) | 94.0 (12.5) | 82.3 (18.8) | ∗∗∗ |
| **Donors/grafts** |  |  |  |  |  |  |  |  |
| **Preoperative** |  |  |  |  |  |  |  |  |
| Age | years | 40.8 (13.4) | 39.4 (12.0) | 37.8 (11.2) | 37.3 (11.2) | 37.8 (9.9) | 38.1 (11.0) | 𝑁.𝑆. |
| Height | cm | 166.9 (7.6) | 167.3 (8.6) | 166.2 (8.2) | 166.7 (8.3) | 165.1 (8.4) | 166.1 (8.3) | 𝑁.𝑆. |
| Weight | kg | 66.5 (11.4) | 63.9 (11.0) | 61.6 (10.3) | 62.3 (10.7) | 60.1 (9.0) | 61.8 (10.2) | ∗ |
| BSA | m^2^ | 1.8 (0.2) | 1.7 (0.2) | 1.7 (0.2) | 1.7 (0.2) | 1.7 (0.2) | 1.7 (0.2) | ∗ |
| BMI | kg/m^2^ | 23.8 (2.9) | 22.8 (3.0) | 22.2 (2.6) | 22.3 (3.0) | 22.0 (2.2) | 22.3 (2.6) | ∗∗ |
| Total liver volume | 10^2^ mL | 1159.0 (201.8) | 1188.7 (219.3) | 1101.8 (196.3) | 1171.4 (213.2) | 1089.6 (163.1) | 1126.6 (196.9) | ∗∗∗ |
| Donor/recipient weight ratio | − | 1.1 (0.2) | 1.1 (0.3) | 1.0 (0.2) | 1.0 (0.2) | 1.0 (0.2) | 1.0 (0.2) | ∗∗∗ |
| Predict GV/SLV | % | 45.7 (11.8) | 46.9 (11.9) | 47.3 (9.8) | 46.8 (9.5) | 46.6 (9.9) | 46.9 (10.2) | 𝑁.𝑆. |
| **During surgery** |  |  |  |  |  |  |  |  |
| Blood loss | mL | 376.9 (255.1) | 535.4 (513.3) | 447.8 (381.4) | 470.4 (368.6) | 267.0 (224.8) | 401.7 (366.5) | ∗∗∗ |
| Surgery time | min | 385.9 (84.8) | 414.3 (88.8) | 372.3 (94.5) | 375.7 (98.2) | 320.1 (76.4) | 362.7 (93.9) | ∗∗∗ |
| Expected residual liver | % | 56.1 (11.1) | 59.0 (11.6) | 52.4 (12.9) | 56.0 (13.3) | 55.0 (12.0) | 55.1 (12.5) | ∗∗ |
| Graft weight | g | 503.2 (123.8) | 475.3 (129.7) | 505.7 (121.0) | 497.5 (124.9) | 476.1 (110.7) | 489.9 (120.3) | 𝑁.𝑆. |
| GV/SLV | % | 42.6 (9.9) | 42.3 (10.7) | 43.5 (9.3) | 41.5 (8.7) | 41.0 (8.6) | 42.1 (9.2) | 𝑁.𝑆. |
| GRWR | − | 0.8 (0.2) | 0.8 (0.2) | 0.8 (0.2) | 0.8 (0.2) | 0.8 (0.2) | 0.8 (0.2) | 𝑁.𝑆. |
| **After surgery** |  |  |  |  |  |  |  |  |
| Max T-BIL | mg/dL | 3.2 (1.8) | 2.4 (1.6) | 2.5 (1.2) | 2.4 (1.2) | 2.1 (0.8) | 2.4 (1.2) | ∗∗∗ |
| Max ALT | U/L | 535.8 (280.4) | 516.0 (296.8) | 481.8 (223.0) | 517.3 (276.0) | 491.8 (222.2) | 499.6 (247.9) | 𝑁.𝑆. |

†: $N.S. :p>0.05$, $* :p\leq0.05$, $** :p\leq0.01$, $*** :p\leq0.001$

**Table C | Comparison of clinical data in each group**

| **Clinical data** | **G1 (N=31)** | **G2 (N=81)** | **G3 (N=161)** | **G4 (N=106)** | **G5 (N=184)** | **Derivation (N=563)** | **Fisher test** |
| --- | --- | --- | --- | --- | --- | --- | --- |
| **Categorical data** |  |  |  |  |  |  |  |
| **Recipients** |  |  |  |  |  |  |  |
| **Preoperative** |  |  |  |  |  |  | ***p*-value^†^** |
| **Diagnosis** |  |  |  |  |  |  | ∗∗∗ |
| Hepatocellular disease | 71.0% | 44.4% | 60.9% | 76.4% | 72.3% | 65.7% |  |
| Cholestasis | 12.9% | 33.3% | 16.1% | 19.8% | 22.8% | 21.3% |  |
| Acute liver failure | 9.7% | 16.0% | 20.5% | 0.0% | 1.1% | 9.1% |  |
| Hepatocellular disease + cholestasis | 0.0% | 0.0% | 0.0% | 0.9% | 0.0% | 0.2% |  |
| metabolic disease | 0.0% | 0.0% | 0.6% | 0.0% | 0.0% | 0.2% |  |
| Other | 6.5% | 6.2% | 1.9% | 1.9% | 3.3% | 3.2% |  |
| **HCC** |  |  |  |  |  |  | ∗∗∗ |
| Yes | 16.1% | 24.7% | 31.1% | 50.9% | 38.0% | 35.3% |  |
| **Sex** |  |  |  |  |  |  | ∗∗ |
| M | 58.1% | 32.1% | 49.1% | 59.4% | 42.4% | 46.9% |  |
| F | 41.9% | 67.9% | 50.9% | 40.6% | 57.6% | 53.1% |  |
| **Gender combination** |  |  |  |  |  |  | ∗∗ |
| Man to man | 38.7% | 21.0% | 31.7% | 38.7% | 19.6% | 27.9% |  |
| Man to woman | 32.3% | 51.9% | 30.4% | 26.4% | 36.4% | 34.8% |  |
| Woman to woman | 9.7% | 16.0% | 20.5% | 14.2% | 21.2% | 18.3% |  |
| Woman to man | 19.4% | 11.1% | 17.4% | 20.8% | 22.8% | 19.0% |  |
| **HBsAb** |  |  |  |  |  |  | ∗∗ |
| Yes | 45.2% | 23.5% | 23.0% | 22.6% | 16.8% | 22.2% |  |
| (+) | 0.0% | 0.0% | 0.0% | 0.0% | 0.0% | 0.0% |  |
| ND | 3.2% | 1.2% | 0.0% | 0.0% | 0.0% | 0.4% |  |
| **HTLV1** |  |  |  |  |  |  | 𝑁.𝑆. |
| Yes | 3.2% | 7.4% | 3.1% | 5.7% | 6.5% | 5.3% |  |
| **HCVAB** |  |  |  |  |  |  | ∗ |
| Yes | 32.3% | 22.2% | 32.9% | 38.7% | 28.8% | 31.1% |  |
| Yes(RNA-) | 0.0% | 2.5% | 0.0% | 3.8% | 3.8% | 2.3% |  |
| **Precondition** |  |  |  |  |  |  | ∗∗∗ |
| Home | 32.3% | 44.4% | 28.6% | 84.9% | 92.9% | 62.7% |  |
| Hospitalized | 45.2% | 32.1% | 49.7% | 15.1% | 7.1% | 26.5% |  |
| ICU | 22.6% | 21.0% | 21.7% | 0.0% | 0.0% | 10.5% |  |
| **Relationship** |  |  |  |  |  |  | 𝑁.𝑆. |
| Brother | 6.5% | 12.3% | 9.3% | 9.4% | 9.2% | 9.6% |  |
| Son | 35.5% | 39.5% | 39.1% | 35.8% | 36.4% | 37.5% |  |
| Parent | 6.5% | 9.9% | 5.0% | 3.8% | 2.2% | 4.6% |  |
| Daughter | 6.5% | 9.9% | 18.0% | 17.9% | 27.2% | 19.2% |  |
| Sister | 9.7% | 6.2% | 5.6% | 6.6% | 6.0% | 6.2% |  |
| Wife | 9.7% | 6.2% | 8.7% | 8.5% | 9.2% | 8.5% |  |
| Husband | 16.1% | 13.6% | 8.1% | 10.4% | 7.1% | 9.4% |  |
| Nephew | 3.2% | 0.0% | 0.6% | 0.9% | 1.1% | 0.9% |  |
| Cousin | 0.0% | 0.0% | 0.0% | 0.9% | 0.5% | 0.4% |  |
| Aunt | 0.0% | 0.0% | 0.6% | 0.0% | 0.0% | 0.2% |  |
| Niece | 0.0% | 0.0% | 0.0% | 0.0% | 0.0% | 0.0% |  |
| Other | 6.5% | 1.2% | 5.0% | 5.7% | 1.1% | 3.4% |  |
| **Blood relative** |  |  |  |  |  |  | 𝑁.𝑆. |
| Yes | 67.7% | 79.0% | 78.3% | 75.5% | 82.6% | 78.7% |  |
| **Blood type** |  |  |  |  |  |  | 𝑁.𝑆. |
| AB Rh(+) | 12.9% | 14.8% | 14.3% | 12.3% | 10.9% | 12.8% |  |
| A Rh(+) | 51.6% | 44.4% | 37.3% | 38.7% | 35.3% | 38.7% |  |
| O Rh(+) | 16.1% | 25.9% | 25.5% | 31.1% | 27.7% | 26.8% |  |
| B Rh(+) | 19.4% | 14.8% | 23.0% | 17.9% | 25.0% | 21.3% |  |
| A Rh(-) | 0.0% | 0.0% | 0.0% | 0.0% | 1.1% | 0.4% |  |
| ABO |  |  |  |  |  |  | ∗∗∗ |
| Identical | 74.2% | 63.0% | 62.7% | 60.4% | 57.1% | 61.1% |  |
| Compatible | 19.4% | 30.9% | 17.4% | 32.1% | 20.1% | 23.1% |  |
| Incompatible | 6.5% | 6.2% | 19.9% | 7.5% | 22.8% | 15.8% |  |
| **Portal vein thrombosis** |  |  |  |  |  |  | 𝑁.𝑆. |
| Yes | 22.6% | 11.1% | 19.3% | 18.9% | 15.2% | 16.9% |  |
| **DM** |  |  |  |  |  |  | ∗∗ |
| Yes | 41.9% | 14.8% | 16.1% | 21.7% | 16.3% | 18.5% |  |
| **Refractory ascites** |  |  |  |  |  |  | ∗∗ |
| 0.0 | 16.1% | 25.9% | 18.6% | 38.7% | 31.5% | 27.5% |  |
| 1.0 | 83.9% | 66.7% | 75.2% | 61.3% | 62.5% | 67.7% |  |
| **CKD** |  |  |  |  |  |  | ∗∗∗ |
| Yes | 25.8% | 22.2% | 28.0% | 15.1% | 12.0% | 19.4% |  |
| **Child class** |  |  |  |  |  |  | ∗∗∗ |
| Child A | 0.0% | 4.9% | 1.2% | 10.4% | 3.8% | 4.3% |  |
| Child B | 3.2% | 14.8% | 5.6% | 30.2% | 25.5% | 17.9% |  |
| Child C | 83.9% | 61.7% | 72.7% | 59.4% | 70.1% | 68.4% |  |
| FHF | 12.9% | 16.0% | 20.5% | 0.0% | 0.5% | 9.1% |  |
| **During surgery** |  |  |  |  |  |  |  |
| **Portal modulation** |  |  |  |  |  |  | ∗∗∗ |
| None | 38.7% | 48.1% | 25.5% | 29.2% | 5.4% | 23.6% |  |
| Splenectomy | 45.2% | 40.7% | 65.2% | 65.1% | 90.2% | 68.7% |  |
| Splenic artery ligation | 16.1% | 8.6% | 9.3% | 5.7% | 4.3% | 7.3% |  |
| **History of upper abdominal laparotomy** |  |  |  |  |  |  | 𝑁.𝑆. |
| Yes | 22.6% | 18.5% | 16.8% | 19.8% | 19.6% | 18.8% |  |
| **Liver resection or liver transplant history** |  |  |  |  |  |  | 𝑁.𝑆. |
| Yes | 3.2% | 4.9% | 3.1% | 3.8% | 4.3% | 3.9% |  |
| **Esophageal varices** |  |  |  |  |  |  | 𝑁.𝑆. |
| Yes | 16.1% | 16.0% | 21.1% | 21.7% | 19.0% | 19.5% |  |
| Yes(treated) | 35.5% | 28.4% | 25.5% | 34.9% | 32.1% | 30.4% |  |
| **Portocaval shunt (>10mm)** |  |  |  |  |  |  | ∗∗ |
| None | 64.5% | 71.6% | 64.6% | 58.5% | 54.9% | 61.3% |  |
| Yes and ligated | 35.5% | 24.7% | 35.4% | 41.5% | 45.1% | 38.2% |  |
| No ligation | 0.0% | 1.2% | 0.0% | 0.0% | 0.0% | 0.2% |  |
| **Veno-venous bypass** |  |  |  |  |  |  | ∗∗. |
| Yes | 22.6% | 9.9% | 10.6% | 12.3% | 3.3% | 9.1% |  |
| **Biliary reconstruction** |  |  |  |  |  |  | ∗∗∗ |
| Roux-en Y | 25.8% | 24.7% | 14.3% | 11.3% | 5.4% | 13.0% |  |
| Duct-to-duct | 74.2% | 72.8% | 85.1% | 88.7% | 94.6% | 86.5% |  |
| **Bile duct suture method** |  |  |  |  |  |  | ∗∗ |
| Interrupted+continuous | 19.4% | 23.5% | 23.6% | 21.7% | 13.0% | 19.5% |  |
| Interrupted | 74.2% | 63.0% | 73.3% | 74.5% | 85.9% | 76.2% |  |
| Continuous | 6.5% | 11.1% | 2.5% | 3.8% | 1.1% | 3.7% |  |
| **Biliary stent** |  |  |  |  |  |  | ∗∗∗ |
| External fistula | 87.1% | 87.7% | 92.5% | 97.2% | 100.0% | 94.8% |  |
| Internal | 3.2% | 4.9% | 1.9% | 0.0% | 0.0% | 1.4% |  |
| **After surgery** |  |  |  |  |  |  |  |
| **CNI** |  |  |  |  |  |  | ∗∗∗ |
| CYA | 19.4% | 22.2% | 24.2% | 28.3% | 12.5% | 20.6% |  |
| FK | 71.0% | 53.1% | 60.9% | 64.2% | 82.6% | 68.0% |  |
| FK→CYA | 9.7% | 12.3% | 9.9% | 1.9% | 2.2% | 6.2% |  |
| CYA→FK | 0.0% | 7.4% | 3.1% | 1.9% | 1.1% | 2.7% |  |
| **MMF induction** |  |  |  |  |  |  | ∗∗∗ |
| Yes | 71.0% | 64.2% | 80.1% | 83.0% | 96.2% | 83.1% |  |
| **Simulect** |  |  |  |  |  |  | ∗∗∗ |
| Yes | 6.5% | 8.6% | 1.9% | 0.0% | 0.5% | 2.3% |  |
| **Bile leak** |  |  |  |  |  |  | ∗∗ |
| Yes | 19.4% | 17.3% | 5.6% | 7.5% | 6.0% | 8.5% |  |
| **Donors / grafts** |  |  |  |  |  |  |  |
| **Preoperative** |  |  |  |  |  |  |  |
| **HBcAb** |  |  |  |  |  |  | 𝑁.𝑆. |
| Yes | 19.4% | 12.3% | 14.9% | 13.2% | 9.8% | 12.8% |  |
| **HTLV1** |  |  |  |  |  |  | 𝑁.𝑆. |
| Yes | 0.0% | 3.7% | 1.2% | 0.9% | 1.1% | 1.4% |  |
| **Sex** |  |  |  |  |  |  | 𝑁.𝑆. |
| M | 71.0% | 72.8% | 62.1% | 65.1% | 56.0% | 62.7% |  |
| F | 29.0% | 27.2% | 37.9% | 34.9% | 44.0% | 37.3% |  |
| **Technique** |  |  |  |  |  |  | ∗∗∗ |
| Right lobectomy | 35.5% | 32.1% | 55.9% | 47.2% | 51.1% | 48.1% |  |
| Extended left lobectomy + caudate lobectomy | 51.6% | 59.3% | 37.3% | 45.3% | 36.4% | 42.5% |  |
| Extended left lobectomy | 3.2% | 6.2% | 5.0% | 1.9% | 1.1% | 3.2% |  |
| Extended right lobectomy | 0.0% | 0.0% | 0.6% | 0.9% | 0.0% | 0.4% |  |
| Extended posterior segmentectomy | 9.7% | 1.2% | 0.0% | 0.9% | 3.3% | 2.0% |  |
| Left lobectomy + caudate lobectomy | 0.0% | 1.2% | 1.2% | 3.8% | 7.6% | 3.7% |  |
| **Blood type** |  |  |  |  |  |  | ∗∗. |
| AB Rh(+) | 9.7% | 3.7% | 14.3% | 3.8% | 10.3% | 9.2% |  |
| A Rh(+) | 41.9% | 39.5% | 37.9% | 32.1% | 35.9% | 36.6% |  |
| O Rh(+) | 35.5% | 44.4% | 29.8% | 49.1% | 29.9% | 35.9% |  |
| B Rh(+) | 9.7% | 12.3% | 18.0% | 14.2% | 23.4% | 17.8% |  |
| A Rh(-) | 3.2% | 0.0% | 0.0% | 0.0% | 0.0% | 0.2% |  |
| **During surgery** |  |  |  |  |  |  |  |
| **Skin incision** |  |  |  |  |  |  | ∗∗∗ |
| Benz | 51.6% | 55.6% | 39.8% | 43.4% | 16.3% | 35.7% |  |
| Reverse L | 6.5% | 14.8% | 17.4% | 14.2% | 13.0% | 14.4% |  |
| Midline | 41.9% | 29.6% | 42.2% | 40.6% | 70.7% | 49.4% |  |
| Midline lap auxiliary | 0.0% | 0.0% | 0.6% | 0.0% | 0.0% | 0.2% |  |
| **After surgery** |  |  |  |  |  |  |  |
| **Complication CD class** |  |  |  |  |  |  | 𝑁.𝑆. |
| I | 9.7% | 14.8% | 16.1% | 9.4% | 12.5% | 13.1% |  |
| II | 9.7% | 6.2% | 8.1% | 9.4% | 8.7% | 8.3% |  |
| IIIa | 9.7% | 0.0% | 3.7% | 5.7% | 6.0% | 4.6% |  |
| IIIb | 0.0% | 2.5% | 1.2% | 1.9% | 1.6% | 1.6% |  |
| **Graft loss** | 87.1% | 38.3% | 19.9% | 19.8% | 9.8% | 22.9% |  |

†: $N.S. :p>0.05$, $* :p\leq0.05$, $** :p\leq0.01$, $*** :p\leq0.00$

**Table D | Comparison of Generalized Wilcoxon test results with Benjamini-Hochberg correction in derivation cohort data**

| **Combination** | ***p*-value** |
| --- | --- |
| G1-G2 | < 0.001 |
| G1-G3 | < 0.001 |
| G1-G4 | < 0.001 |
| G1-G5 | < 0.001 |
| G2-G3 | 0.005 |
| G2-G4 | 0.006 |
| G2-G5 | <0.001 |
| G3-G4 | 0.814 |
| G3-G5 | 0.356 |
| G4-G5 | 0.369 |

**Table E | Comparison of Bonferroni-corrected Wilcoxon rank sum test** **results**

| **Clinical data** | **G1-G2** | **G1-G3** | **G1-G4** | **G1-G5** | **G2-G3** | **G2-G4** | **G2-G5** | **G3-G4** | **G3-G5** | **G4-G5** |
| --- | --- | --- | --- | --- | --- | --- | --- | --- | --- | --- |
| **Numeric data** |  |  |  |  |  |  |  |  |  |  |
| **Recipients** |  |  |  |  |  |  |  |  |  |  |
| **Preoperative** |  |  |  |  |  |  |  |  |  |  |
| Mono | 0.701 | >0.99 | >0.99 | >0.99 | >0.99 | >0.99 | >0.99 | >0.99 | >0.99 | >0.99 |
| NLR | >0.99 | >0.99 | <0.001 | <0.001 | 0.051 | <0.001 | <0.001 | <0.001 | <0.001 | 0.099 |
| LMR | 0.270 | >0.99 | <0.001 | <0.001 | <0.001 | <0.001 | 0.003 | <0.001 | <0.001 | 0.013 |
| Age | >0.99 | >0.99 | >0.99 | >0.99 | 0.388 | 0.352 | 0.009 | >0.99 | >0.99 | >0.99 |
| Height | >0.99 | >0.99 | >0.99 | >0.99 | 0.923 | 0.040 | >0.99 | >0.99 | >0.99 | 0.243 |
| Weight | 0.121 | >0.99 | >0.99 | >0.99 | 0.021 | <0.001 | 0.032 | >0.99 | >0.99 | >0.99 |
| BMI | 0.837 | >0.99 | >0.99 | >0.99 | 0.139 | 0.011 | 0.014 | >0.99 | >0.99 | >0.99 |
| BSA | 0.083 | >0.99 | >0.99 | >0.99 | 0.023 | <0.001 | 0.093 | >0.99 | >0.99 | 0.845 |
| T-BIL | 0.351 | >0.99 | <0.001 | <0.001 | 0.977 | <0.001 | 0.001 | <0.001 | <0.001 | 0.633 |
| Albumin | >0.99 | >0.99 | >0.99 | >0.99 | >0.99 | >0.99 | >0.99 | >0.99 | 0.411 | >0.99 |
| PT | >0.99 | >0.99 | 0.014 | 0.001 | >0.99 | 0.004 | <0.001 | <0.001 | <0.001 | >0.99 |
| APTT | >0.99 | >0.99 | >0.99 | 0.134 | >0.99 | >0.99 | 0.102 | 0.004 | <0.001 | 0.763 |
| BUN | >0.99 | >0.99 | 0.002 | <0.001 | >0.99 | 0.035 | <0.001 | 0.009 | <0.001 | >0.99 |
| Cr | >0.99 | >0.99 | >0.99 | 0.331 | >0.99 | >0.99 | 0.085 | 0.054 | <0.001 | >0.99 |
| GFR | >0.99 | >0.99 | >0.99 | >0.99 | >0.99 | 0.388 | 0.126 | 0.021 | 0.001 | >0.99 |
| Na | 0.528 | >0.99 | 0.003 | 0.002 | 0.516 | 0.626 | 0.429 | <0.001 | <0.001 | >0.99 |
| WBC | >0.99 | >0.99 | 0.544 | >0.99 | >0.99 | <0.001 | 0.010 | <0.001 | <0.001 | >0.99 |
| PLT | >0.99 | >0.99 | >0.99 | >0.99 | >0.99 | >0.99 | >0.99 | >0.99 | 0.853 | 0.698 |
| Hb | >0.99 | 0.718 | <0.001 | <0.001 | 0.969 | <0.001 | <0.001 | 0.001 | <0.001 | >0.99 |
| **During surgery** |  |  |  |  |  |  |  |  |  |  |
| Surgery time | >0.99 | >0.99 | >0.99 | 0.014 | >0.99 | >0.99 | 0.002 | >0.99 | 0.006 | 0.019 |
| Blood loss | 0.035 | 0.177 | 0.001 | <0.001 | >0.99 | >0.99 | 0.002 | 0.019 | <0.001 | 0.312 |
| Warm ischemic time | 0.092 | >0.99 | 0.938 | >0.99 | 0.118 | 0.579 | 0.035 | >0.99 | >0.99 | >0.99 |
| Cold ischemic time | 0.059 | >0.99 | >0.99 | >0.99 | 0.032 | 0.118 | <0.001 | >0.99 | 0.874 | >0.99 |
| Portal blood flow/GW | >0.99 | >0.99 | >0.99 | >0.99 | >0.99 | >0.99 | >0.99 | >0.99 | >0.99 | >0.99 |
| Post-reperfusion portal pressure | >0.99 | >0.99 | >0.99 | >0.99 | 0.068 | >0.99 | >0.99 | 0.264 | 0.110 | >0.99 |
| Final portal pressure | >0.99 | 0.006 | 0.078 | 0.011 | 0.027 | >0.99 | 0.015 | >0.99 | >0.99 | >0.99 |
| **After surgery** |  |  |  |  |  |  |  |  |  |  |
| T-BIL (14POD) | <0.001 | <0.001 | <0.001 | <0.001 | <0.001 | <0.001 | <0.001 | 0.027 | <0.001 | 0.002 |
| Ascites (14POD) | >0.99 | <0.001 | <0.001 | <0.001 | <0.001 | <0.001 | <0.001 | >0.99 | <0.001 | <0.001 |
| Ascites (30POD) | 0.516 | <0.001 | <0.001 | <0.001 | <0.001 | <0.001 | <0.001 | >0.99 | 0.042 | >0.99 |
| PT (14POD) | >0.99 | <0.001 | 0.001 | <0.001 | <0.001 | <0.001 | <0.001 | <0.001 | <0.001 | <0.001 |
| **Donors/grafts** |  |  |  |  |  |  |  |  |  |  |
| **Preoperative** |  |  |  |  |  |  |  |  |  |  |
| Age | >0.99 | >0.99 | >0.99 | >0.99 | >0.99 | >0.99 | >0.99 | >0.99 | >0.99 | >0.99 |
| Height | >0.99 | >0.99 | >0.99 | >0.99 | >0.99 | >0.99 | 0.629 | >0.99 | >0.99 | >0.99 |
| Weight | >0.99 | 0.231 | 0.820 | 0.042 | 0.848 | >0.99 | 0.085 | >0.99 | >0.99 | >0.99 |
| BSA | >0.99 | 0.583 | >0.99 | 0.137 | >0.99 | >0.99 | 0.105 | >0.99 | >0.99 | >0.99 |
| BMI | >0.99 | 0.038 | 0.210 | 0.007 | 0.409 | >0.99 | 0.099 | >0.99 | >0.99 | >0.99 |
| Total liver volume | >0.99 | >0.99 | >0.99 | 0.776 | 0.027 | >0.99 | 0.008 | 0.092 | >0.99 | 0.025 |
| Donor/recipient weight ratio | >0.99 | >0.99 | >0.99 | >0.99 | 0.010 | 0.003 | <0.001 | >0.99 | >0.99 | >0.99 |
| Predict GV/SLV | >0.99 | >0.99 | >0.99 | >0.99 | >0.99 | >0.99 | >0.99 | >0.99 | >0.99 | >0.99 |
| **During surgery** |  |  |  |  |  |  |  |  |  |  |
| Blood loss | >0.99 | >0.99 | >0.99 | 0.053 | >0.99 | >0.99 | <0.001 | >0.99 | <0.001 | <0.001 |
| Surgery time | >0.99 | >0.99 | >0.99 | 0.001 | 0.007 | 0.100 | <0.001 | >0.99 | <0.001 | <0.001 |
| Expected residual liver | >0.99 | >0.99 | >0.99 | >0.99 | 0.001 | >0.99 | 0.110 | 0.163 | 0.613 | >0.99 |
| Graft weight | >0.99 | >0.99 | >0.99 | >0.99 | 0.368 | >0.99 | >0.99 | >0.99 | 0.150 | >0.99 |
| GV/SLV | >0.99 | >0.99 | >0.99 | >0.99 | >0.99 | >0.99 | >0.99 | 0.960 | 0.072 | >0.99 |
| GRWR | >0.99 | >0.99 | >0.99 | >0.99 | >0.99 | >0.99 | 0.588 | 0.452 | 0.124 | >0.99 |
| **After surgery** |  |  |  |  |  |  |  |  |  |  |
| Max T-BIL | 0.009 | 0.118 | 0.043 | <0.001 | 0.639 | >0.99 | >0.99 | >0.99 | 0.003 | 0.897 |
| Max ALT | >0.99 | >0.99 | >0.99 | >0.99 | >0.99 | >0.99 | >0.99 | >0.99 | >0.99 | >0.99 |

**Table F | Comparison of Bonferroni-corrected Fisher's exact test results**

| **Clinical data** | **G1-G2** | **G1-G3** | **G1-G4** | **G1-G5** | **G2-G3** | **G2-G4** | **G2-G5** | **G3-G4** | **G3-G5** | **G4-G5** |
| --- | --- | --- | --- | --- | --- | --- | --- | --- | --- | --- |
| **Categorical data** |  |  |  |  |  |  |  |  |  |  |
| **Recipients** |  |  |  |  |  |  |  |  |  |  |
| **Preoperative** |  |  |  |  |  |  |  |  |  |  |
| Diagnosis |  |  |  |  |  |  |  |  |  |  |
| Hepatocellular disease | 0.193 | >0.99 | >0.99 | >0.99 | 0.197 | <0.001 | <0.001 | 0.112 | 0.292 | >0.99 |
| Cholestasis | 0.348 | >0.99 | >0.99 | >0.99 | 0.030 | 0.431 | 0.941 | >0.99 | >0.99 | >0.99 |
| Acute liver failure | >0.99 | >0.99 | 0.107 | 0.223 | >0.99 | <0.001 | <0.001 | <0.001 | <0.001 | >0.99 |
| Hepatocellular disease + cholestasis | >0.99 | >0.99 | >0.99 | >0.99 | >0.99 | >0.99 | >0.99 | >0.99 | >0.99 | >0.99 |
| Metabolic disease | >0.99 | >0.99 | >0.99 | >0.99 | >0.99 | >0.99 | >0.99 | >0.99 | >0.99 | >0.99 |
| Other | >0.99 | >0.99 | >0.99 | >0.99 | >0.99 | >0.99 | >0.99 | >0.99 | >0.99 | >0.99 |
| HCC |  |  |  |  |  |  |  |  |  |  |
| Yes | >0.99 | >0.99 | 0.008 | 0.237 | >0.99 | 0.003 | 0.358 | 0.013 | >0.99 | 0.365 |
| Sex |  |  |  |  |  |  |  |  |  |  |
| M | 0.170 | >0.99 | >0.99 | >0.99 | 0.135 | 0.002 | >0.99 | >0.99 | >0.99 | 0.071 |
| Gender combination |  |  |  |  |  |  |  |  |  |  |
| Man to man | 0.897 | >0.99 | >0.99 | 0.332 | 0.959 | 0.108 | >0.99 | >0.99 | 0.127 | 0.005 |
| Man to woman | 0.897 | >0.99 | >0.99 | >0.99 | 0.019 | 0.005 | 0.215 | >0.99 | >0.99 | 0.918 |
| Woman to woman | >0.99 | >0.99 | >0.99 | >0.99 | >0.99 | >0.99 | >0.99 | >0.99 | >0.99 | >0.99 |
| Woman to man | >0.99 | >0.99 | >0.99 | >0.99 | >0.99 | >0.99 | 0.280 | >0.99 | >0.99 | >0.99 |
| HBsAb |  |  |  |  |  |  |  |  |  |  |
| Yes | 0.362 | 0.146 | 0.215 | 0.012 | >0.99 | >0.99 | >0.99 | >0.99 | >0.99 | >0.99 |
| (+) | >0.99 | >0.99 | >0.99 | >0.99 | >0.99 | >0.99 | >0.99 | >0.99 | >0.99 | >0.99 |
| ND | >0.99 | >0.99 | >0.99 | >0.99 | >0.99 | >0.99 | >0.99 | >0.99 | >0.99 | >0.99 |
| HTLV1 |  |  |  |  |  |  |  |  |  |  |
| Yes | >0.99 | >0.99 | >0.99 | >0.99 | >0.99 | >0.99 | >0.99 | >0.99 | >0.99 | >0.99 |
| HCVAB |  |  |  |  |  |  |  |  |  |  |
| Yes | >0.99 | >0.99 | >0.99 | >0.99 | >0.99 | 0.178 | >0.99 | >0.99 | >0.99 | 0.914 |
| Yes(RNA-) | >0.99 | >0.99 | >0.99 | >0.99 | >0.99 | >0.99 | >0.99 | 0.240 | 0.161 | >0.99 |
| Precondition |  |  |  |  |  |  |  |  |  |  |
| Home | >0.99 | >0.99 | <0.001 | <0.001 | 0.151 | <0.001 | <0.001 | <0.001 | <0.001 | 0.406 |
| Hospitalized | >0.99 | >0.99 | 0.009 | <0.001 | 0.095 | 0.077 | <0.001 | <0.001 | <0.001 | 0.406 |
| ICU | >0.99 | >0.99 | <0.001 | <0.001 | >0.99 | <0.001 | <0.001 | <0.001 | <0.001 | >0.99 |
| Relationship |  |  |  |  |  |  |  |  |  |  |
| Brother | >0.99 | >0.99 | >0.99 | >0.99 | >0.99 | >0.99 | >0.99 | >0.99 | >0.99 | >0.99 |
| Son | >0.99 | >0.99 | >0.99 | >0.99 | >0.99 | >0.99 | >0.99 | >0.99 | >0.99 | >0.99 |
| Parent | >0.99 | >0.99 | >0.99 | >0.99 | >0.99 | >0.99 | 0.092 | >0.99 | >0.99 | >0.99 |
| Daughter | >0.99 | >0.99 | >0.99 | 0.117 | >0.99 | >0.99 | 0.013 | >0.99 | 0.538 | 0.862 |
| Sister | >0.99 | >0.99 | >0.99 | >0.99 | >0.99 | >0.99 | >0.99 | >0.99 | >0.99 | >0.99 |
| Wife | >0.99 | >0.99 | >0.99 | >0.99 | >0.99 | >0.99 | >0.99 | >0.99 | >0.99 | >0.99 |
| Husband | >0.99 | >0.99 | >0.99 | >0.99 | >0.99 | >0.99 | >0.99 | >0.99 | >0.99 | >0.99 |
| Nephew | >0.99 | >0.99 | >0.99 | >0.99 | >0.99 | >0.99 | >0.99 | >0.99 | >0.99 | >0.99 |
| Cousin | >0.99 | >0.99 | >0.99 | >0.99 | >0.99 | >0.99 | >0.99 | >0.99 | >0.99 | >0.99 |
| Aunt | >0.99 | >0.99 | >0.99 | >0.99 | >0.99 | >0.99 | >0.99 | >0.99 | >0.99 | >0.99 |
| Niece | >0.99 | >0.99 | >0.99 | >0.99 | >0.99 | >0.99 | >0.99 | >0.99 | >0.99 | >0.99 |
| Other | >0.99 | >0.99 | >0.99 | >0.99 | >0.99 | >0.99 | >0.99 | >0.99 | 0.498 | 0.546 |
| Blood relative |  |  |  |  |  |  |  |  |  |  |
| Yes | >0.99 | >0.99 | >0.99 | 0.829 | >0.99 | >0.99 | >0.99 | >0.99 | >0.99 | >0.99 |
| Blood type |  |  |  |  |  |  |  |  |  |  |
| AB Rh(+) | >0.99 | >0.99 | >0.99 | >0.99 | >0.99 | >0.99 | >0.99 | >0.99 | >0.99 | >0.99 |
| A Rh(+) | >0.99 | >0.99 | >0.99 | >0.99 | >0.99 | >0.99 | >0.99 | >0.99 | >0.99 | >0.99 |
| O Rh(+) | >0.99 | >0.99 | >0.99 | >0.99 | >0.99 | >0.99 | >0.99 | >0.99 | >0.99 | >0.99 |
| B Rh(+) | >0.99 | >0.99 | >0.99 | >0.99 | >0.99 | >0.99 | 0.763 | >0.99 | >0.99 | >0.99 |
| A Rh(-) | >0.99 | >0.99 | >0.99 | >0.99 | >0.99 | >0.99 | >0.99 | >0.99 | >0.99 | >0.99 |
| ABO |  |  |  |  |  |  |  |  |  |  |
| Identical | >0.99 | >0.99 | >0.99 | 0.784 | >0.99 | >0.99 | >0.99 | >0.99 | >0.99 | >0.99 |
| Compatible | >0.99 | >0.99 | >0.99 | >0.99 | 0.211 | >0.99 | 0.607 | 0.074 | >0.99 | 0.329 |
| Incompatible | >0.99 | >0.99 | >0.99 | 0.512 | 0.044 | >0.99 | 0.008 | 0.052 | >0.99 | 0.007 |
| Portal vein thrombosis 2 |  |  |  |  |  |  |  |  |  |  |
| Yes | >0.99 | >0.99 | >0.99 | >0.99 | >0.99 | >0.99 | >0.99 | >0.99 | >0.99 | 0.996 |
| DM |  |  |  |  |  |  |  |  |  |  |
| Yes | 0.043 | 0.026 | 0.358 | 0.026 | >0.99 | >0.99 | >0.99 | >0.99 | >0.99 | >0.99 |
| Refractory ascites |  |  |  |  |  |  |  |  |  |  |
| 1.0 | >0.99 | >0.99 | 0.293 | 0.239 | >0.99 | >0.99 | >0.99 | 0.206 | 0.146 | >0.99 |
| CKD |  |  |  |  |  |  |  |  |  |  |
| Yes | >0.99 | >0.99 | >0.99 | 0.505 | >0.99 | >0.99 | 0.402 | 0.169 | 0.002 | >0.99 |
| Child class |  |  |  |  |  |  |  |  |  |  |
| Child A | >0.99 | >0.99 | 0.694 | >0.99 | 0.987 | >0.99 | >0.99 | 0.009 | >0.99 | 0.404 |
| Child B | >0.99 | >0.99 | 0.014 | 0.042 | 0.270 | 0.153 | 0.560 | <0.001 | <0.001 | >0.99 |
| Child C | 0.259 | >0.99 | 0.175 | >0.99 | >0.99 | >0.99 | >0.99 | 0.324 | >0.99 | 0.718 |
| FHF | >0.99 | >0.99 | 0.022 | 0.016 | >0.99 | <0.001 | <0.001 | <0.001 | <0.001 | >0.99 |
| **During surgery** |  |  |  |  |  |  |  |  |  |  |
| Portal modulation |  |  |  |  |  |  |  |  |  |  |
| Splenectomy | >0.99 | 0.436 | 0.600 | <0.001 | 0.003 | 0.011 | <0.001 | >0.99 | <0.001 | <0.001 |
| Splenic artery ligation | >0.99 | >0.99 | >0.99 | 0.250 | >0.99 | >0.99 | >0.99 | >0.99 | 0.831 | >0.99 |
| History of upper abdominal laparotomy |  |  |  |  |  |  |  |  |  |  |
| Yes | >0.99 | >0.99 | >0.99 | >0.99 | >0.99 | >0.99 | >0.99 | >0.99 | >0.99 | >0.99 |
| Liver resection or liver transplant history |  |  |  |  |  |  |  |  |  |  |
| Yes | >0.99 | >0.99 | >0.99 | >0.99 | >0.99 | >0.99 | >0.99 | >0.99 | >0.99 | >0.99 |
| Esophageal varices |  |  |  |  |  |  |  |  |  |  |
| Yes | >0.99 | >0.99 | >0.99 | >0.99 | >0.99 | >0.99 | >0.99 | >0.99 | >0.99 | >0.99 |
| Yes(treated) | >0.99 | >0.99 | >0.99 | >0.99 | >0.99 | >0.99 | >0.99 | >0.99 | >0.99 | >0.99 |
| Portocaval shunt (>10mm) |  |  |  |  |  |  |  |  |  |  |
| Yes and ligated | >0.99 | >0.99 | >0.99 | >0.99 | >0.99 | 0.197 | 0.017 | >0.99 | 0.787 | >0.99 |
| No ligation | >0.99 | >0.99 | >0.99 | >0.99 | >0.99 | >0.99 | >0.99 | >0.99 | >0.99 | >0.99 |
| Veno-venous bypass |  |  |  |  |  |  |  |  |  |  |
| Yes | >0.99 | 0.767 | >0.99 | 0.006 | >0.99 | >0.99 | 0.365 | >0.99 | 0.086 | 0.053 |
| Biliary reconstruction |  |  |  |  |  |  |  |  |  |  |
| Roux-en Y | >0.99 | >0.99 | 0.780 | 0.012 | 0.514 | 0.192 | <0.001 | >0.99 | 0.059 | >0.99 |
| Duct-to-duct | >0.99 | >0.99 | 0.780 | 0.012 | 0.250 | 0.071 | <0.001 | >0.99 | 0.036 | >0.99 |
| Suture method |  |  |  |  |  |  |  |  |  |  |
| Interrupted+continuous | >0.99 | >0.99 | >0.99 | >0.99 | >0.99 | >0.99 | 0.458 | >0.99 | 0.117 | 0.683 |
| Interrupted | >0.99 | >0.99 | >0.99 | >0.99 | >0.99 | >0.99 | <0.001 | >0.99 | 0.045 | 0.184 |
| Continuous | >0.99 | >0.99 | >0.99 | >0.99 | 0.117 | 0.789 | 0.005 | >0.99 | >0.99 | >0.99 |
| Biliary stent |  |  |  |  |  |  |  |  |  |  |
| External fistula | >0.99 | >0.99 | 0.461 | 0.004 | >0.99 | 0.176 | <0.001 | >0.99 | <0.001 | 0.480 |
| Internal | >0.99 | >0.99 | >0.99 | >0.99 | >0.99 | 0.337 | 0.083 | >0.99 | >0.99 | >0.99 |
| **After surgery** |  |  |  |  |  |  |  |  |  |  |
| CNI |  |  |  |  |  |  |  |  |  |  |
| CYA | >0.99 | >0.99 | >0.99 | >0.99 | >0.99 | >0.99 | 0.638 | >0.99 | 0.050 | 0.014 |
| FK | 0.930 | >0.99 | >0.99 | >0.99 | >0.99 | >0.99 | <0.001 | >0.99 | <0.001 | 0.006 |
| FK→CYA | >0.99 | >0.99 | 0.763 | 0.636 | >0.99 | 0.053 | 0.015 | 0.112 | 0.023 | >0.99 |
| CYA→FK | >0.99 | >0.99 | >0.99 | >0.99 | >0.99 | 0.787 | 0.113 | >0.99 | >0.99 | >0.99 |
| MMF induction |  |  |  |  |  |  |  |  |  |  |
| Yes | >0.99 | >0.99 | >0.99 | <0.001 | 0.116 | 0.039 | <0.001 | >0.99 | <0.001 | 0.003 |
| Simulect |  |  |  |  |  |  |  |  |  |  |
| Yes | >0.99 | >0.99 | 0.499 | 0.551 | 0.180 | 0.025 | 0.012 | >0.99 | >0.99 | >0.99 |
| Bile leak |  |  |  |  |  |  |  |  |  |  |
| Yes | >0.99 | 0.189 | 0.861 | 0.215 | 0.051 | 0.649 | 0.058 | >0.99 | >0.99 | >0.99 |
| **Donors/grafts** |  |  |  |  |  |  |  |  |  |  |
| **Preoperative** |  |  |  |  |  |  |  |  |  |  |
| HBcAb |  |  |  |  |  |  |  |  |  |  |
| Yes | >0.99 | >0.99 | >0.99 | >0.99 | >0.99 | >0.99 | >0.99 | >0.99 | >0.99 | >0.99 |
| HTLV1 |  |  |  |  |  |  |  |  |  |  |
| Yes | >0.99 | >0.99 | >0.99 | >0.99 | >0.99 | >0.99 | >0.99 | >0.99 | >0.99 | >0.99 |
| Sex |  |  |  |  |  |  |  |  |  |  |
| M | >0.99 | >0.99 | >0.99 | >0.99 | >0.99 | >0.99 | 0.097 | >0.99 | >0.99 | >0.99 |
| Technique |  |  |  |  |  |  |  |  |  |  |
| Right lobectomy | >0.99 | 0.490 | >0.99 | >0.99 | 0.006 | 0.505 | 0.049 | >0.99 | >0.99 | >0.99 |
| Extended left lobectomy + caudate lobectomy | >0.99 | >0.99 | >0.99 | >0.99 | 0.016 | 0.763 | 0.007 | >0.99 | >0.99 | >0.99 |
| Extended left lobectomy | >0.99 | >0.99 | >0.99 | >0.99 | >0.99 | >0.99 | 0.294 | >0.99 | 0.498 | >0.99 |
| Extended right lobectomy | >0.99 | >0.99 | >0.99 | >0.99 | >0.99 | >0.99 | >0.99 | >0.99 | >0.99 | >0.99 |
| Extended posterior segmentectomy | 0.637 | 0.039 | 0.362 | >0.99 | >0.99 | >0.99 | >0.99 | >0.99 | 0.320 | >0.99 |
| Left lobectomy + caudate lobectomy | >0.99 | >0.99 | >0.99 | >0.99 | >0.99 | >0.99 | 0.432 | >0.99 | 0.046 | >0.99 |
| Blood type |  |  |  |  |  |  |  |  |  |  |
| AB Rh(+) | >0.99 | >0.99 | >0.99 | >0.99 | 0.142 | >0.99 | 0.908 | 0.061 | >0.99 | 0.688 |
| A Rh(+) | >0.99 | >0.99 | 0.765 | >0.99 | >0.99 | >0.99 | >0.99 | >0.99 | >0.99 | >0.99 |
| O Rh(+) | >0.99 | >0.99 | >0.99 | >0.99 | 0.316 | >0.99 | 0.250 | 0.019 | >0.99 | 0.015 |
| B Rh(+) | >0.99 | >0.99 | >0.99 | >0.99 | >0.99 | >0.99 | 0.453 | >0.99 | >0.99 | 0.677 |
| A Rh(-) | >0.99 | >0.99 | >0.99 | >0.99 | >0.99 | >0.99 | >0.99 | >0.99 | >0.99 | >0.99 |
| **During surgery** |  |  |  |  |  |  |  |  |  |  |
| Skin incision |  |  |  |  |  |  |  |  |  |  |
| Benz | >0.99 | >0.99 | >0.99 | <0.001 | 0.208 | >0.99 | <0.001 | >0.99 | <0.001 | <0.001 |
| Reverse L | >0.99 | >0.99 | >0.99 | >0.99 | >0.99 | >0.99 | >0.99 | >0.99 | >0.99 | >0.99 |
| Midline | >0.99 | >0.99 | >0.99 | 0.034 | 0.682 | >0.99 | <0.001 | >0.99 | <0.001 | <0.001 |
| Midline lap auxiliary | >0.99 | >0.99 | >0.99 | >0.99 | >0.99 | >0.99 | >0.99 | >0.99 | >0.99 | >0.99 |
| **After surgery** |  |  |  |  |  |  |  |  |  |  |
| Complication CD class |  |  |  |  |  |  |  |  |  |  |
| I | >0.99 | >0.99 | >0.99 | >0.99 | >0.99 | >0.99 | >0.99 | >0.99 | >0.99 | >0.99 |
| II | >0.99 | >0.99 | >0.99 | >0.99 | >0.99 | >0.99 | >0.99 | >0.99 | >0.99 | >0.99 |
| IIIa | 0.197 | >0.99 | >0.99 | >0.99 | >0.99 | 0.371 | 0.205 | >0.99 | >0.99 | >0.99 |
| IIIb | >0.99 | >0.99 | >0.99 | >0.99 | >0.99 | >0.99 | >0.99 | >0.99 | >0.99 | >0.99 |

**Table G | Comparison of Generalized Wilcoxon test results with Benjamini-Hochberg correction in group-annotated validation cohort data**

| **Proposed classification** |  |  |  |
| --- | --- | --- | --- |
|  | **Early-loss* vs Intermediate-loss*** | **Early-loss* vs Late/No-loss*** | **Intermediate-loss* vs Late/No-loss*** |
| Multi-label | 𝑁.𝑆.^†^ | 𝑁.𝑆. | ∗ |
| Hierarchical | ∗ | ∗∗∗ | ∗ |
| **Conventional classification** |  |  |  |
|  | **PG1**^‡^ **vs PG2** | **PG1 vs PG3** | **PG2 vs PG3** |
| predictive score | 𝑁.𝑆. | 𝑁.𝑆. | 𝑁.𝑆. |

†: $N.S. :p>0.05$, $* :p\leq0.05$, $** :p\leq0.01$, $*** :p\leq0.001$

‡: PG1: Group with predictive score less than 1.15; PG2: Group with predictive score between 1.15 and 1.30; PG3: Group with predictive score of 1.30 or higher.
