# Supplementary material for: Prediction of graft loss in living donor liver transplantation during the early postoperative period
Source: PLoS Comput Biol. 2025 Nov 24;21(11):e1013734. doi: 10.1371/journal.pcbi.1013734 (PMC12671733; doi:10.1371/journal.pcbi.1013734)
Supplement: Attachment — Submitted filename: PBP_revision_RY.pdf [file pcbi.1013734.s003.pdf]

Response to reviewer comments

Raiki Yoshimura, Naotoshi Nakamura, Takeru Matsuura, Takeo Toshima, Takasuke Fukuhara, Kazuyuki Aihara, Katsuhito Fujiu, Shingo Iwami, and Tomoharu Yoshizumi

October 8, 2025

We would like to thank the reviewers for their helpful comments and suggestions. In this document, we present the reviewers' comments in black and provide our responses in red. All changes made in response to the reviewers' comments on PCOMPBIOL-D-25-00261 have been incorporated in the revised version of the manuscript. All coauthors have agreed with these changes and have approved the final submitted version of the manuscript.

Editor comments:

Thank you for submitting your manuscript to PLOS Computational Biology. After careful consideration, we feel that it has merit but does not fully meet PLOS Computational Biology's publication criteria as it currently stands. Therefore, we invite you to submit a revised version of the manuscript that addresses the points raised during the review process.

Reply to Editor comments:

We appreciate the constructive feedback and suggestions from the three reviewers to improve the quality of our manuscript. Below is a list of the revised files, along with a brief description of the major changes made to each one.

1. Manuscript file
    - Revisions have been made to the **Abstract**, **Author Summary**, **Introduction**, **Results**, **Discussion**, and **Methods** (see the replies to the comments below for details).
    - **Tables 1**, **2**, **5**, and **6** replace **Figs 4**, **5**, and **10,** respectively
    - **Tables 3** and **4** are new

2. Supplementary Information
    - **Fig S1** and **Fig S2** are new

Reviewer #1:

The authors predict graft loss in living donor liver transplantation. This is an interesting interdisciplinary piece of work.

As I am ML specialist, my review will touch ML issues primarily. I consider the ms almost ready to publish, however, I would like to double-check, that the cross validation proceedure was done correctly. I assume it was, but want to be 100% sure and therefore ask for clarification.

1. Please confirm and clearly write in the ms, that only data from the training set was used for standardization, imputation, hyperparameter tuning etc. In the ms the training data set is denoted as derivation data set.
   If data from the validation data set is used in any of standardization, imputation, hyperparameter tuning etc then the results would be flawed.

Reply to comment 1:

Thank you for this comment. We confirm that the standardization, imputation, and hyperparameter tuning procedure were conducted using data from the derivation cohort only. To clarify this point, we have added sentences in the **Data preparation** (lines 513- 517) and **Hyperparameter tuning** (lines 541-542) sections of the **Methods**, respectively.

2. In line 209 - 211 The sentence: 'Note that in this model ... ' is unclear in this respect.

Reply to comment 2:

Thank you for pointing out this ambiguity. Patient stratification into groups was conducted using only the derivation dataset; the validation dataset was not involved in this process.

Since group labels were not assigned to the validation dataset, it could not be used to evaluate group prediction performance.

Instead, we assessed the prediction performance using 4-fold cross-validation within the derivation dataset. We used the default XGBoost settings without hyperparameter tuning. For preprocessing, the scaler and KNN imputer were fitted on each training fold and applied to the corresponding test fold to prevent data leakage. To apply the model to the validation dataset, we trained

the scaler, imputer, and classifier on the full derivation dataset and applied them to the validation data.

To clarify this point, we have added sentences in the **Multilabel classification** section of the **Methods** (lines 643-648).

3. l 527 ' then the best parameters...'

Reply to comment 3:

We confirm that hyperparameter tuning was performed **using the derivation dataset only**, and the validation dataset was not used in this process. To clarify this point, we have added a detailed description of the tuning procedure and have explicitly noted that the validation dataset was not used, in the **Hyperparameter tuning** subsection of the **Methods** (lines 541-557).

4. typo l 432 computed tomography

Reply to comment 4:

Thank you for the comment. We appreciate your pointing out what appeared to be a typographical error. However, we used the term ‘computed tomography’ to reflect our methodology of assessing graft volume through CT scans. We sincerely appreciate your taking the time to carefully review our work.

5. Finally, I have a question. In order to use the model in practice, it is important that for an individual patient the prediction is correct. Could you perhaps discuss, whether the model allows to assess uncertainty so that a wrong classification can be avoided...

Reply to comment 5:

Thank you for this important comment. As you correctly point out, ensuring accurate classification for individual patients is crucial for the safe clinical use of our model, especially to avoid false negatives in high-risk cases.

One approach to reducing the risk of misclassification is to adjust the probability threshold at which patients are classified as high risk. For example, by identifying a threshold that achieves a recall of ≥0.95, we can minimize the likelihood of overlooking patients who will experience early graft loss. However, this approach often comes at the cost of reduced precision—leading to an

increased number of patients being flagged as high risk, resulting in unnecessary clinical burden or increased cost.

To address this trade-off, we conducted a Decision Curve Analysis (**Fig S2**). The results indicate that setting the threshold within the range of 0.05 to 0.15 yields a net benefit greater than 0.01, suggesting this range is optimal from a clinical utility perspective.

Nonetheless, misclassifications are still possible. Therefore, from a clinical standpoint, we recommend that model predictions be used as supportive tools in conjunction with physician judgment, rather than as standalone criteria. Particularly in borderline cases, integrating the model's risk estimate with clinical context—such as signs of small-for-size syndrome (SFSS), infection risk, or delayed recovery—can help to ensure safer and more individualized decision-making. We have added this to the **Results** section (lines 147-153).

Reviewer #2:

This study retrospectively analyzed data from 748 LDLT patients and applied machine learning to predict early graft loss within 180 days postoperatively, outperforming traditional models. A hierarchical prediction approach was developed to identify high- and intermediate-risk patients (G1 and G2) within 30 days after surgery, enabling timely clinical interventions to improve transplant outcomes.

The authors have done a lot of work in the field of living donor liver transplantation, but my main points of view are as follows.

1. In the "Significance Statement," it is recommended to further emphasize the practical implications of this study for clinical decision-making, particularly how the model can guide early interventions.

Reply to comment 1:

Thank you very much for your suggestion. In response, we have revised the "**Significance Statement**" (now titled "**Author Summary**" in the revised manuscript) to better highlight the clinical relevance of our findings. Specifically, we emphasize that timely risk stratification shortly after LDLT allows clinicians to:

(1) promptly consider critical steps such as DDLT registration or re-transplant evaluation for high-risk patients at risk for early graft loss and
(2) initiate intensified monitoring and management for patients in the intermediate-loss group (e.g., early detection of biliary complications or recurrence).

By clearly linking these predictive outputs to concrete clinical actions, our study highlights the direct utility of the model in guiding early interventions and improving patient outcomes. We have added these points to the revised manuscript (lines 29-32).

2. In the "Main Text," it would strengthen the rationale for developing LDLT-specific predictive models to include a brief literature review comparing the mechanisms and postoperative risks of LDLT versus DDLT.

Reply to comment 2:

Thank you for your valuable suggestion. In response, we have revised the Main Text to include a brief comparison of LDLT and DDLT in terms of graft failure mechanisms and postoperative complications.

Unlike DDLT, LDLT frequently involves partial grafts, which increases the risk of graft dysfunction, particularly small-for-size syndrome (SFSS). In LDLT, graft volume is often constrained by donor safety considerations, leading to higher portal pressure and shear stress, both of which contribute to hepatocellular injury and early graft loss [1]. Moreover, the biliary anatomy in LDLT poses a unique challenge: the bile duct is typically only 2–5 mm in diameter, increasing the technical difficulty of anastomosis and making patients more susceptible to biliary complications such as anastomotic strictures [2]. These complications occur more commonly in LDLT than in DDLT and are associated with significant morbidity and potential graft loss [3]. Therefore, it is essential to develop LDLT-specific prediction models that reflect these distinct clinical and anatomical challenges. We have added this discussion to the revised manuscript (lines 44-51).

3. The data presented in Table S4 do not conclusively demonstrate that the RF classifier is the optimal model, as its performance is not consistently superior to the others. We recommend including more detailed descriptions of the cross-validation procedures to enhance the rigor of the model selection process.

Reply to comment 3:

Thank you for your comments. As shown in Table 1, although models such as XGBoost and LightGBM demonstrated similarly high predictive performance, the Random Forest (RF) classifier achieved the highest ROC-AUC among them. While this slight performance advantage contributed to our selection, the decision was primarily motivated by the structural characteristics of the model, which are particularly suited for downstream analyses.

Specifically, XGBoost and LightGBM are gradient boosting algorithms that sequentially construct dependent trees by correcting errors from previous iterations. In contrast, RF builds each decision tree independently using bootstrapped datasets. This independence enables the computation of proximity-based distance matrices. Although this is also possible with boosting methods,

RF offers greater robustness and representational capacity, making it more suitable for capturing inter-patient similarity in stratification tasks. Thus, the structural properties of RF were critical not only for predictive modeling, but also for enabling subsequent analyses based on inter-patient similarity.

Regarding model selection, we performed five-fold cross-validation within the derivation cohort and optimized hyperparameters using GridSearchCV. This procedure is described in the **Methods** section.

Based on both predictive accuracy and downstream stratification performance, we therefore chose the RF classifier for these analyses.

We have added this justification to the revised manuscript (lines 561-564)

4. Although seven key predictors were identified by SHAP analysis, we suggest specifying clinical threshold values or abnormal ranges for each variable to improve interpretability and applicability in clinical settings.

Reply to comment 4:

Thank you for the important suggestion. To improve the interpretability and clinical applicability of the SHAP-selected predictors, we have now plotted standard clinical reference ranges for each of the important variables in the comparison plots between the early graft loss and non-loss groups (see revised **Fig 1F**). This visualization clearly demonstrates that patients with early graft loss tend to show greater deviations from normal ranges, particularly in variables such as prothrombin time activity (%), serum total bilirubin, and postoperative ascites volume. For clarity, we have also included annotations in the figure legend indicating the standard reference ranges.

5. The statement “however, we found that donor and graft information did not significantly contribute to the prediction of early graft loss” warrants further discussion. We recommend exploring potential clinical explanations for this finding.

Reply to comment 5:

Thank you for the comment. We have now added a brief discussion to address this point. While previous studies based on univariate or simple multivariate analyses have reported that factors such as donor age and graft weight are associated with post-transplant outcomes, our comprehensive

machine learning–based approach revealed that donor and graft variables had limited overall predictive power for early graft loss. This likely reflects the high consistency in graft quality at our high-volume LDLT center, where donor selection criteria and surgical techniques are strictly standardized. Moreover, in LDLT, cold ischemia time is uniformly short, further reducing variability.

Importantly, this overall finding does not preclude the possibility that donor- or graft-related factors may contribute to graft loss in specific subgroups. For instance, as discussed in our response to the next **comment 6**, subgroup G1 exhibited clinical features suggestive of marginal graft function or perioperative injury, implying that these variables may still play a role under certain conditions.

This interpretation has been incorporated into the revised **Discussion** section (lines 384–389).

6. The analysis indicating that G2 closely resembles G1 in characteristics but exhibits different survival outcomes would benefit from mechanistic insights into the clinical distinctions between these two groups.

Reply to comment 6:

Thank you for your comment. Although G1 (early-loss group) and G2 (intermediate-loss group) share several perioperative characteristics, our data suggest that the mechanisms underlying graft loss in these two groups are distinct, which likely accounts for their differing clinical trajectories.

In the early-loss subgroup, early graft loss appears to be driven primarily by factors related to graft quality and perioperative injury. Specifically, recipients in the early-loss subgroup exhibited significantly higher total bilirubin (T-Bil) levels on postoperative day 14 compared with the intermediate-loss subgroup, and notably, donors in the early-loss subgroup also had elevated postoperative T-Bil levels (see **Fig S4** and **Fig A**). These findings suggest that the grafts in the early-loss subgroup were more vulnerable, potentially due to suboptimal donor liver quality or early cholestatic injury. Additionally, early-loss patients had greater intraoperative blood loss and slightly longer cold ischemic times, both of which are known to exacerbate ischemia-reperfusion injury and to contribute to early graft dysfunction (see **Table S4** and **Fig A**).

Taken together, we hypothesize that early-loss represents a phenotype of early graft failure primarily due to perioperative insult and marginal graft quality,

whereas intermediate-loss represents a trajectory of delayed graft loss stemming from post-transplant complications after an initially stable recovery.

This subgroup-specific interpretation complements our overall findings described in the response to <u>the previous **comment 5**</u>, where donor and graft factors were found to have limited predictive power across the entire cohort but may still influence outcomes in selected cases.

We have incorporated this mechanistic interpretation into the revised **Discussion** section (lines 405-412).

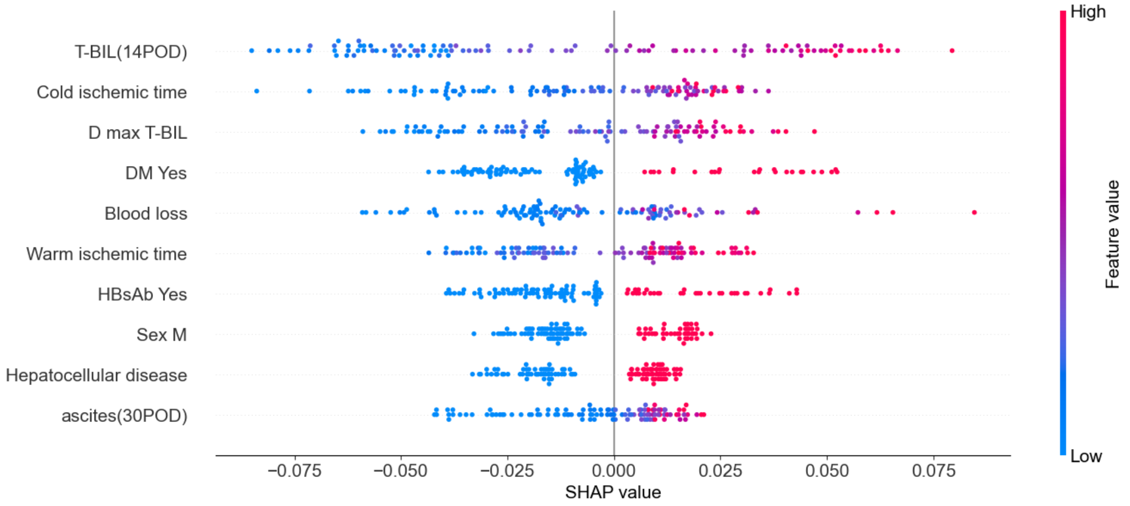

Fig A Visualization of the contribution in the G1–G2 binary classification using SHAP

7. In the "Group-Level Prediction" section, we recommend including confusion matrices or more comprehensive classification metrics such as F1-scores to better assess model performance across the three binary classification tasks.

Reply to comment 7:

Thank you for this helpful suggestion. In response, we have added a new table (**Table 4**) that presents additional classification metrics—including the F1-score, precision, and recall—for each of the three binary classification tasks in the group-level prediction.

8. We suggest a more in-depth discussion of the reasons behind the poor performance of the multilabel classification model, including specific evidence that low recall for G1 may be due to the small sample size.

Reply to comment 8:

Thank you for the comment. We have added further discussion to explore the limitations of the multilabel classification model.

As you pointed out, the low recall for early-loss patients (G1) is likely attributable to the small sample size in the validation cohort (n = 29), which limits the model's ability to capture subgroup-specific patterns.

In addition, patients with late or no graft loss (G3, G4, G5) appear to constitute a clinically and statistically distinct population compared to those with early (G1) or intermediate graft loss (G2). This is supported by the model's higher performance in binary classification tasks that distinguish late/no-loss patients from G1 or G2, as shown in **Fig. 2D** and **Table S7**.

Consequently, during multilabel learning, the model may have disproportionately focused on separating the more clearly defined late/no-loss (G3+G4+G5) group, while underfitting the more subtle boundary between early-loss (G1) and intermediate-loss (G2).

This interpretation has been added to the revised **Discussion** section (lines 403-405).

9. To enhance the argument for this study's contribution, we recommend a systematic comparison of the proposed model's performance with existing LDLT prediction models, ideally supported by statistical significance testing.

Reply to comment 9:

Thank you for this constructive suggestion. While statistical comparison is essential for demonstrating the added value of our proposed model, it is important to note that existing models specifically developed for LDLT are extremely limited, as LDLT is primarily performed in Asia and is not widely studied in Western transplant settings. As such, most conventional models were not originally designed for LDLT populations. Among the few available, we performed a statistical comparison of our proposed model against existing LDLT prediction models, including the D-MELD, predictive score, and eGLR. Using DeLong's test for pairwise comparison of ROC-AUC values, we found that our model significantly outperformed all baseline models ($p < 0.01$ for all comparisons in the validation cohort). These results support the added predictive value of our machine learning–based approach. We have added corresponding p-values in **Table 2**.

10. As the discussion mentions that changes in surgical techniques and immunosuppressive regimens may affect prediction accuracy, we suggest including corresponding trend plots or descriptive analyses of these variables over time.

Reply to comment 10:

Thank you for the helpful suggestion. We have now added a descriptive analysis to clarify the potential influence of surgical and immunosuppressive trends over time. In particular, we observed a gradual increase in the use of right lobe grafts and changes in hepatic vein reconstruction methods starting around 2004 and 2011, respectively. However, the distribution of surgical years across the stratified groups (G1–G5) was relatively uniform, suggesting that temporal bias was minimal.

As for immunosuppressive agents, none were identified as significant predictors of early graft loss in our model. This may be explained by the fact that immunosuppressive protocols−including calcineurin inhibitors, mycophenolate mofetil, and steroids−have been relatively standardized at our institution throughout the study period. These considerations have been added to the revised **Discussion** section (lines 455-460).

11. Regarding future work, please discuss the possibility of applying advanced graph learning techniques (10.1109/TNSE.2024.3524077) for improved performance.

Reply to comment 11:

Thank you for the suggestion regarding the application of advanced graph learning techniques. Those approaches are indeed promising for capturing complex relational structures in medical data. While our current dataset does not include detailed network-level information, such as donor-recipient familial relationships or omics-based biological connections, we agree that incorporating such data in future work could provide a suitable foundation for applying graph-based models. We consider this an exciting direction for enhancing prediction performance and uncovering deeper mechanistic insights.

This interpretation has been added to the **Discussion** section (lines 442-444).

Reviewer #3:

The study is based on data from a single, high-volume center (Kyushu University Hospital), which may limit its generalizability. Variations in patient demographics, surgical practices, and perioperative management across institutions could affect the model's performance elsewhere.

Additionally, the external validation cohort is relatively small, and the number of early graft loss events (n=12 in the validation set) is particularly limited. This may compromise the statistical power and robustness of performance evaluation for the classification models, especially for rare outcomes. However, other concerns are included below.

Reply to general remark:

Thank you very much for pointing out this important limitation. We fully acknowledge that conducting the study at a single high-volume LDLT center (Kyushu University Hospital) may limit the generalizability of our findings, given potential differences in patient demographics, surgical techniques, and perioperative management across institutions.

That said, we would like to note that our institution serves as a referral center for LDLT and receives patients from a broad range of hospitals across Japan. This may help to mitigate, to some extent, selection bias in patient characteristics.

To evaluate the robustness of our model within the current dataset, we conducted multiple random train-test splits using different seeds and confirmed consistent predictive accuracy. Additionally, cross-validation within the derivation cohort was performed to support the internal validity of our findings.

We fully agree that external validation is crucial for assessing the broader applicability of our model. In light of this, we are currently preparing to validate our model using independent datasets from other transplant centers. We have revised the **Discussion** section to explicitly address this limitation and to describe our ongoing efforts toward external validation (lines 424-430).

1. Further clarification is needed on how the identified features can be interpreted clinically. For instance, guidance on actionable thresholds or how clinicians should adjust care based on model outputs is minimal.

Reply to comment 1:

Thank you for your valuable comment. We agree that improving the clinical interpretability and usability of the model outputs is essential for practical application.

As our model is based on machine learning, which captures complex and nonlinear interactions among variables, it is inherently difficult to define explicit, universal thresholds for each predictor. To assist with interpretation, we have included reference (normal) ranges for selected variables in **Fig. 2F**, which may help readers contextualize the clinical relevance of the observed deviations.

To facilitate more practical bedside use, we are currently developing a simplified clinical risk score derived from the top six features identified in this study. This score, designed to enhance transparency and usability, is under development and will be reported in a future study.

In its current form, the model stratifies patients into three risk categories—early, intermediate, and late/no graft loss—based on predicted group assignment (G1, G2, G3–G5). These categories may help guide clinical decision-making. For example, patients predicted to be at high risk of early graft loss may benefit from intensified monitoring, early consideration of re-transplantation, or referral for deceased donor liver transplantation. Intermediate-risk patients may require closer surveillance for complications such as biliary strictures or disease recurrence, while standard follow-up may suffice for low-risk patients unless new concerns arise.

Importantly, we recommend that model predictions can be used as supportive tools, not as standalone criteria. Particularly in borderline cases, integrating the model's risk estimates with clinical judgment—considering factors such as small-for-size syndrome (SFSS), infection risk, or delayed postoperative recovery—can support more nuanced and individualized patient care.

These points have been added to the revised **Discussion** section (lines 463-465).

2. The prediction of long-term graft loss using only early postoperative data showed limited success (ROC-AUC 0.59–0.78). The authors acknowledge this but might further elaborate on strategies to improve this (e.g., incorporating longitudinal follow-up data). Add calibration plots and decision curve analysis to evaluate clinical utility beyond ROC-AUC.

Reply to comment 2:

Thank you for your comment. As noted, long-term graft loss was more difficult to predict based on early postoperative data alone. To improve this, we are currently preparing to incorporate longitudinal follow-up data from routine postoperative check-ups, as well as data from other transplant centers. We plan to evaluate whether the inclusion of such temporal information enhances long-term prognostic accuracy in future work. We have added this point to the revised **Discussion** section (lines 442-444).

To address your suggestion regarding clinical utility, we have added both calibration plots and decision curve analysis (DCA) for the early graft loss model (see **Fig S2**). The calibration plot demonstrates that the model is generally well-calibrated but slightly underestimates risk overall. The final segment of the curve drops sharply due to the small number of patients with predicted probabilities above 0.5 (only two cases). The DCA suggests a positive net benefit in the threshold probability range of approximately 0.10–0.15, supporting its potential clinical usefulness. We have added this point to the revised **Results** section (lines 147-153).

3. The grouping into G1–G5 and then merging into G1, G2, and G3+G4+G5 may be confusing to readers. A more intuitive naming convention or visual summary could improve clarity.

Reply to comment 3:

Thank you for the helpful suggestion. To improve clarity and make the grouping more intuitive, we have replaced the original labels (G1, G2, and G3+G4+G5) with clinically meaningful terms: **early-loss**, **intermediate-loss**, and **late/no-loss**. These new labels are used consistently throughout the revised manuscript and figures.

4. The authors uses k-NN imputation for missing values. Given the high dimensionality, the choice and validation of this method need more justification, as different imputation strategies might influence model performance.

Reply to comment 4:

Thank you for your important point regarding the choice of imputation strategy. We initially selected k-nearest neighbor (k-NN) imputation due to its simplicity and ability to preserve local structure in the data. To evaluate the robustness of our results to different imputation strategies, we additionally tested mean imputation, multiple imputation by chained equations (MICE), and random forest–based imputation. We found that the ROC-AUC remained similar across imputation methods (see **Fig B**). These findings support the stability of our model and are described in the revised **Methods** sections (lines 517-520).

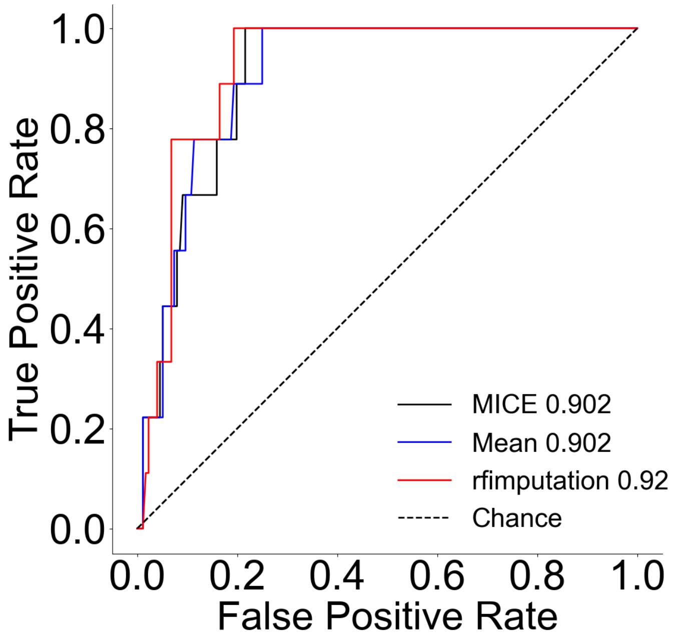

Fig B | Comparison of ROC curves for different imputation methods:
The ROC curve for RF classifiers trained to predict early graft loss (within 180 days post-LDLT) is shown alongside models with different imputation methods for comparison.

5. Improve the discussion of model limitations and future directions, particularly for real-world deployment.

Reply to comment 5:

We sincerely appreciate your insightful comment. We have addressed this point in our ***Reply to comment 1***. Please see the revised Discussion section (lines 463–465) for further details.

6. The absence of true external validation (e.g., from an independent institution or dataset) restricts the model's demonstrated generalizability.

Reply to comment 6:

We sincerely appreciate your insightful comment. As we mentioned in our response to ***Reply to general remark***, we recognize that external validation is crucial for evaluating the generalizability of our model. To that end, we are in the process of preparing external validation using datasets from other transplant centers, which we plan to include in our future work.

7. The number of early graft loss events (especially in the validation set) is quite small relative to the number of features considered, even after SHAP-based reduction. This raises concerns about overfitting and the reliability of model performance metrics.

Reply to comment 7:

Thank you for the comment. We agree that the limited number of early graft loss events poses challenges. To address this, we performed cross-validation within the derivation cohort and tested multiple random train-test splits. The results of these tests were consistent, which helps to mitigate overfitting concerns. These points are addressed in our ***Reply to general remark*** and are reflected in the revised **Discussion** section.

8. Important confounders, such as socioeconomic factors, nutritional status, or long-term immunosuppressive management, were not mentioned or adjusted for. These may impact graft loss outcomes and should be considered or at least discussed.

Reply to comment 8:

Thank you for the thoughtful comment. Indeed, socioeconomic background, nutritional status, and long-term immunosuppressive management are important factors that could influence graft outcomes. However, these variables were not available in our retrospective dataset and could not be included in the current analysis. We acknowledge this as a limitation and have mentioned this in the revised manuscript (lines 444-448).

As for potential confounding variables present in our dataset, we note that tree-based machine learning models such as Random Forest are generally robust against the influence of confounders in prediction tasks due to their hierarchical structure and feature splitting mechanism. Nonetheless, we recognize that confounding can affect the interpretation of feature importance. To mitigate this, we calculated a correlation matrix prior to feature selection and removed features with pairwise correlations above 0.7. As a result, *precondition home*, which was strongly correlated with other clinical indicators, was excluded, while the remaining six variables were retained as important predictors. This additional step strengthens the interpretability of our model while minimizing confounding bias in feature selection (lines 598-600).

9. While the manuscript mentions use of GridSearchCV for hyperparameter optimization, it lacks detail on the grid ranges tested, scoring metrics used for selection, or regularization strategies (e.g., cross-validation techniques to avoid overfitting).

Reply to comment 9:

Thank you for pointing out the need for clarification regarding our hyperparameter tuning procedure. We used GridSearchCV from the scikit-learn library with 5-fold cross-validation on the derivation cohort for model optimization. The primary scoring metric for model selection was ROC-AUC. Grid search ranges for key hyperparameters (e.g., number of estimators, maximum depth, and minimum samples split) were defined as follows:

- RandomForest
    - n_estimators: [10,50,100,500,1000]
    - max_depth: [None,10,20]
    - min_sample_split: [2,5,10]

- XGBoost
    - eta: [0.01, 0.1, 1.0]
    - gamma: [0, 0.1]
    - n_estimators: [10,50,100, 500,1000]
    - max_depth: [None, 10, 20]
    - min_child_weight: [1, 2]

- lightGBM
    - n_estimators: [10,50,100,1000],
    - max_depth: [4,8,16],
    - num_leaves: [31,15,7,3],
    - learning_rate: [0.1,0.05,0.01]

We have added this information to the revised **Methods** section (lines 540-557)

10. SMOTE was used, but the manuscript lacks a discussion on potential pitfalls of synthetic data generation, such as introducing noise or inflating minority class performance unrealistically.

Reply to comment 10:

Thank you for this important point. While SMOTE was employed to address class imbalance in the derivation cohort during model training, we fully acknowledge its limitations, including the risk of introducing synthetic noise or overestimating performance for the minority class. To mitigate this, SMOTE was applied only within the training data and not during model evaluation. All reported performance metrics, including ROC-AUC and precision-recall, were computed using independent validation data without synthetic augmentation. We have added this clarification and a discussion of SMOTE-related limitations to the revised **Discussion** section (lines 424-428).

11. Further clinical interpretation—especially why some features (like preoperative home status or drainage ascites) are strong predictors—is underdeveloped.

Reply to comment 11:

Thank you for this valuable comment. We have expanded the discussion to provide further clinical interpretation of key predictive features identified by the model. For example, preoperative home status reflects the patient's functional reserve and overall condition before LDLT. Patients who were hospitalized or in the ICU before transplantation often present with sarcopenia, poor nutritional status, and increased vulnerability to postoperative complications such as

atelectasis or pneumonia, which can negatively impact graft outcomes. Likewise, increased ascites on postoperative days 14 or 30 may indicate the presence of small-for-size syndrome (SFSS), a complication more specific to LDLT that results from insufficient graft volume relative to portal inflow. SFSS is associated with portal hypertension, cholestasis, coagulopathy, and ultimately, early graft dysfunction. These clinical insights have been added to the revised **Results** and **Discussion** to clarify the mechanistic relevance of these predictors (lines 172-175, 405-412).

However, in the revised manuscript, we excluded *precondition home* from the final important feature, as it was judged to be a strong confounder potentially overlapping with other variables such as preoperative functional status. Importantly, the model's predictive performance remained nearly unchanged, and six features—excluding *precondition home* from the original seven—were ultimately selected.

12. Several key tables and figures critical to understanding the results are placed in the supplementary section (e.g., Tables S1–S6, Fig S1–S3). Consider moving the most important ones to the main manuscript to improve accessibility.

Reply to comment 12:

We appreciate the reviewer's suggestion. To improve clarity and the accessibility of key findings, we have moved the following materials from the supplementary section to the main manuscript:

- **Table 1** (formerly Supplementary Table 4): Comparison of performance for early graft loss prediction among four machine learning models.
- **Table 2** (formerly Supplementary Table 5): Comparison with previously published clinical metrics.
- **Table 3** (newly inserted): Comparison of performance for long-term graft loss prediction among four machine learning models
- **Table 4** (newly inserted): Comparison of binary classifier performance among four machine learning models for early-loss, intermediate-loss, and late/no-loss.

- **Table 5** (formerly Supplementary Table 10): Comparison of multi-class classifier performance among four machine learning models for early-loss, intermediate-loss, and late/no-loss.
- **Table 6** (formerly Supplementary Table 10): Comparison of hierarchical classifier performance among four machine learning models for early-loss, intermediate-loss, and late/no-loss.

Other supporting tables and figures remain in the supplementary section to maintain clarity and avoid overloading the main text.

13. While the model shows high discrimination (ROC-AUC), no clinically meaningful cutoffs, thresholds, or risk bands are proposed. These are essential for translating predictive outputs into actionable decisions.

Reply to comment 13:

Thank you for this important comment. Rather than establishing fixed clinical thresholds for each variable—which may be inappropriate given the nonlinear nature of machine learning models—we aim to implement the trained model as a **web-based clinical decision support tool**. This application will allow physicians to input postoperative values and receive individualized predictions of early, intermediate, or late/no graft loss risk. The predicted group classification can support real-time clinical decisions. For example, patients predicted as having early loss may require intensified monitoring or re-transplant planning; prediction of intermediate loss warrants surveillance for complications, whereas standard follow-up may suffice for late/no-loss cases unless concerns emerge.

We believe this model-level implementation, as opposed to the use of single-variable cutoffs, provides a more comprehensive and realistic approach to clinical integration. Details on this planned deployment have been added to the revised **Discussion** section (lines 463–465).

References

1. Amol Vijay Kanetkar, D.B., Sudhindran Sudhindran, Puneet Dhar, Unnikrishnan Gopalakrishnan, Ramachandran Menon, Othiyil Vayoth Sudheer, *Is Portal Venous Pressure or Porto-systemic Gradient Really A Harbinger of Poor Outcomes After Living Donor Liver Transplantation?* Journal of Clinical and Experimental Hepatology, 2017. **7**: p. 235-246.
2. Cardenas, D.B.M.N.A., *Current management of biliary complications after liver transplantation: Emphasis on endoscopic therapy.* Gastroenterología y Hepatología, 2010. **34**(2): p. 107-115.
3. Giri, S., et al., *Comparison of Biliary Complications Between Living and Deceased Donor Liver Transplantations: A Systematic Review and Meta-analysis.* Cureus Journal of Medical Science, 2024. **16**(9).
